# Sinomenine Inhibits the Progression of Rheumatoid Arthritis by Regulating the Secretion of Inflammatory Cytokines and Monocyte/Macrophage Subsets

**DOI:** 10.3389/fimmu.2018.02228

**Published:** 2018-09-26

**Authors:** Weiwei Liu, Yajie Zhang, Weina Zhu, Chunhua Ma, Jie Ruan, Hongyan Long, Yue Wang

**Affiliations:** ^1^The Affiliated Hospital of Nanjing University of Chinese Medicine, Jiangsu Province Hospital of Traditional Chinese Medicine, Nanjing, China; ^2^Central Laboratory, Nanjing Hospital of Chinese Medicine, The Third Affiliated Hospital of Nanjing University of Chinese Medicine, Nanjing, China; ^3^Clinical Biobank of Nanjing Hospital of Chinese Medicine, Nanjing Hospital of Chinese Medicine, The Third Affiliated Hospital of Nanjing University of Chinese Medicine, Nanjing, China; ^4^Department of Pediatrics, Nanjing Hospital of Chinese Medicine, The Third Affiliated Hospital of Nanjing University of Chinese Medicine, Nanjing, China; ^5^The First Clinical Medical School, Nanjing University of Chinese Medicine, Nanjing, China

**Keywords:** sinomenine, cytokine, inflammatory, rheumatoid arthritis, macrophages, monocytes

## Abstract

Rheumatoid arthritis (RA) is a chronic autoimmune inflammatory arthropathy associated with articular damage and attendant comorbidities. Even although RA treatment has advanced remarkably over the last decade, a significant proportion of patients still do not achieve sustained remission. The cause of RA is not yet known despite the many potential mechanisms proposed. It has been confirmed that RA is associated with dysregulated immune system and persistent inflammation. Therefore, management of inflammation is always the target of therapy. Sinomenine (SIN) is the prescription drug approved by the Chinese government for RA treatment. A previous study found that SIN was a robust anti-inflammation drug. In this study, we screened the different secretory cytokines using inflammation antibody arrays and qRT-PCR in both LPS-induced and SIN-treated RAW264.7 cells followed by evaluation of the ability of SIN to modulate cytokine secretion in a cell model, collagen-induced arthritis (CIA) mouse model, and RA patients. Several clinical indexes affecting the 28-joint disease activity score (DAS28) were determined before and after SIN treatment. Clinical indexes, inflammatory cytokine secretion, and DAS28 were compared among RA patients treated with either SIN or methotrexate (MTX). To explore the mechanism of SIN anti-inflammatory function, RA-associated monocyte/macrophage subsets were determined using flow cytometry in CIA mouse model and RA patients, both treated with SIN. The results demonstrated that SIN regulated IL-6, GM-CSF, IL-12 p40, IL-1α, TNF-α, IL-1β, KC (CXCL1), Eotaxin-2, IL-10, M-CSF, RANTES, and MCP-1 secretion *in vivo* and *in vitro* and reduced RA activity and DAS28 in a clinical setting. Furthermore, SIN attenuated CD11b^+^F4/80^+^CD64^+^ resident macrophages in the synovial tissue, CD11b^+^Ly6C^+^CD43^+^ macrophages in the spleen and draining lymph nodes of CIA mice. The percentage of CD14^+^CD16^+^ peripheral blood mononuclear cells was reduced by SIN in RA patients. These data indicated that SIN regulates the secretion of multiple inflammatory cytokines and monocyte/macrophage subsets, thereby suppressing RA progression. Therefore, along with MTX, SIN could be an alternative cost-effective anti-inflammatory agent for treating RA.

## Introduction

Inflammation, also known as general immune response, is a double-edged sword (1). While it predominantly serves a protective response for the clearance and repair of injured tissues or deteriorating stimuli, dysregulation of an inflammatory response may lead to occurrence of chronic inflammation (2–4). Such chronic, low-grade inflammatory conditions may fester over a long time period and adversely contribute to the development of diseases associated with aging, including rheumatoid arthritis (RA) (5–8). RA, a chronic and systemic autoimmune disease affecting around 0.5–2% of the human population, is characterized by a deforming symmetrical polyarthritis varying in extent and severity, leading to irreversible cartilage and bone damage (9–12).

The initiating cause of RA has not been fully understood yet, but dysregulated immune system has been confirmed to play a major role in the propagation of the disease (12). The various pro-inflammatory cytokines secreted by infiltrating macrophages as well as T and B cells in the synovial fluids and tissues contribute to joint inflammation (13, 14). Among the cytokines, those promoting inflammatory cascades are considered pro-inflammatory mediators, such as interleukin IL-1β, IL-6, IL-8, tumor necrosis factor alpha (TNF-α), chemokine, and interferon families (15). Thus, targeting the reduction of these pro-inflammatory mediators can be an effective way for controlling and preventing chronic inflammatory diseases (8).

Macrophages play a pivotal role in the induction and progression of inflammatory processes by acting as the first line of defense against invading agents (bacteria, viruses, and fungi), responding to pathogenic attacks, such as infection, and performing tumor and immune regulatory functions (16, 17). Prolonged activation of macrophages results in a dysregulated inflammatory response via the release of various pro-inflammatory cytokines (e.g., IL-1, IL-1β, IL-6, IL-8, and TNF-α) and inflammatory mediators, leading to a vicious cycle of chronic inflammation (17).

Methotrexate (MTX) is a disease modifying anti-rheumatic drug (DMARD) commonly used for RA treatment owing to its proven efficacy, relative safety, and cost-effectiveness (18). However, similar to other DMARDs, MTX has its side effects. It can cause rashes and stomach upset, liver or bone marrow toxicity, and birth defects (19). In rare cases, it can also cause dyspnea (20). Therefore, attention has been focused on plant-derived natural compounds as potential candidate drugs for RA treatment owing to their high efficacy and relatively less side effects (21).

Sinomenine (SIN; 7,8-didehydro-4-hydroxy-3,7-dimethoxy-17-methylmorphinan-6-one,CAS Number: 115-53-7, Figure 1A), the main active ingredient in the roots and stems of the plant *Sinomenium acutum* (Thunb.) Rehder & E.H. Wilson (Family *Menispermaceae*), has been utilized to clinically treat rheumatic and arthritic diseases for over 1,000 years by ancient Chinese physicians (22, 23). Interestingly, we found in our previous study that SIN had a better clinical efficacy and fewer adverse events (ADEs) during RA treatment in the clinical setting than MTX therapy (24).

**Figure 1:**
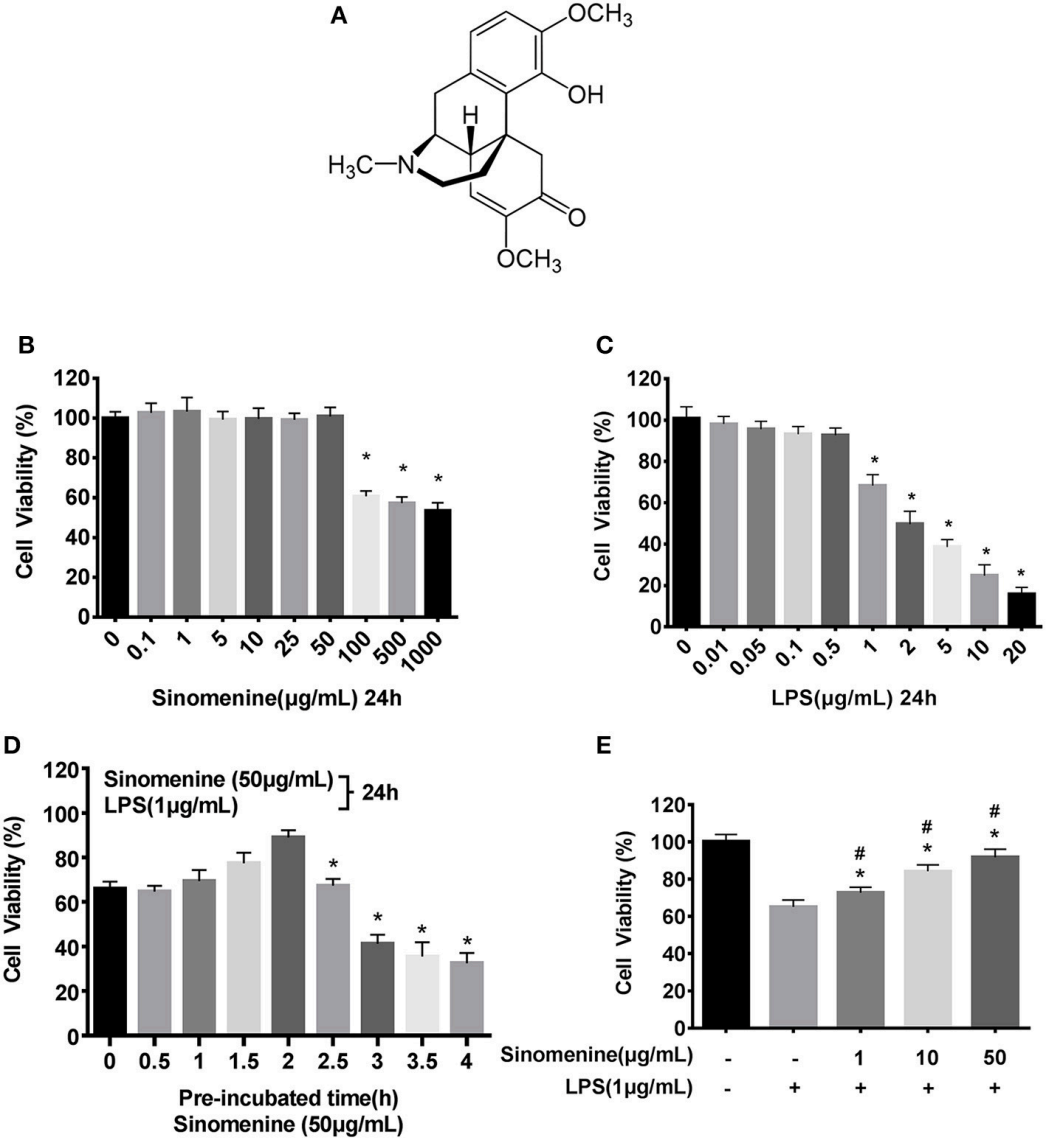
SIN prevents LPS-induced cell injury. Cell viability was determined by the CCK-8 assay, and the viability of untreated RAW264.7 was assessed as 100%. **(A)** Chemical structure of Sinomenine (SIN), CAS number: 115-53-7. **(B)** RAW264.7 cells were incubated with SIN (0–1000 μg/mL) for 24 h. **(C)** RAW264.7 cells were treated with various concentrations of LPS (0–20 μg/mL) for 24 h (*P < 0.05 vs. control). **(D)** RAW264.7 cells were pre-treated with 50 μg/mL SIN for 0–4 h and then co-stimulated with LPS (1 μg/mL) for another 24 h (*P < 0.05 vs. 2 h pre-treated). **(E)** RAW264.7 cells were pre-incubated with 0, 1, 10, 50 μg/mL SIN for 2 h and co-stimulated with 1 μg/mL LPS for another 24 h. Data are presented as mean ± SD values of four independent experiments (*P < 0.05 vs. control, ^#^*P* < 0.05 vs. LPS treated RAW264.7 cells).

Actually, SIN has attracted much interest for its safety profile and strong anti-inflammatory and immune-regulatory properties (25–27). Currently, it has been developed into a series of Chinese proprietary medicines called Zhengqing Fengtongning (ZQFTN) for treating RA and other autoimmune diseases in China. Some specific cytokines and mediators, such as TNF-α and IL-1β, were shown to be attenuated by SIN (28). The relationship between the anti-inflammatory effect of SIN and RA progression has been observed in cell or animal models of arthritis. However, no large-scale study has been conducted to assess the anti-inflammatory effects of SIN on cytokines in both cell and animal models or to assess its therapeutic effects on cytokines in a clinical setting. Therefore, further research would be valuable.

Our current study was undertaken to investigate the potential anti-inflammatory activities of SIN *in vitro* and *in vivo*, especially, its clinical therapeutic effects. First, we analyzed the effect of SIN on LPS-stimulated RAW264.7 cells by measuring the expression levels of 40 cytokines and chemokines that are known to participate in inflammatory progression. Twelve SIN mediated cytokines were discovered using the inflammation antibody array followed by validation with enzyme linked immunosorbent assay (ELISA) and quantitative real-time polymerase chain reaction (qRT-PCR). Furthermore, we observed symptom remission in collagen-induced arthritic mice treated with SIN and analyzed cytokine-associated immune cell subsets in an animal model under SIN administration. Moreover, the therapeutic effects and cytokine secretion in RA patients treated with SIN or MTX were analyzed and assessed, and the relationship between cytokine expression levels and prognosis of RA was explored. In addition, the percentage of CD14^+^CD16^+^ monocytes in peripheral blood mononuclear cells (PBMCs) was compared between SIN-treated and MTX-treated patients. In summary, we elucidated the mechanism of SIN in the treatment of RA from a broader perspective, helping to develop a complementary therapeutic strategy for RA.

## Materials and methods

### Reagent

Sinomenine (SIN; ≥98% high-performance liquid chromatography (HPLC) purity, CAS number: 115-53-7, Figure 1A) and LPS (*Escherichia coli*, O55:B5, EINECS: 297-473-0) were purchased from Sigma-Aldrich Chemical Co. (St. Louis, MO, USA). All reagents used for cell culture contained penicillin, streptomycin, fetal bovine serum (FBS) [purchased from Gibco Life Technologies (Grand Island, NY, USA)], together with Dulbecco's modified Eagle medium (DMEM) and phosphate buffer saline (PBS). Cell Counting Kit-8 (CCK-8) was obtained from Dojindo Molecular Technologies (Shanghai, China). TRIzol® reagents and primers used for qRT-PCR were obtained from Invitrogen (Carlsbad, CA, USA) (see Supplementary materials and methods for primers; Table S1). ELISA kits were purchased from RayBiotech (Norcross, GA, USA) and R&D Systems (Minneapolis, MN, USA) (see Supplementary materials and methods; Table S2).

### Cell line

The murine macrophage-like cell line (RAW264.7) was obtained from the American Type Culture Collection (ATCC, Rockville, MD, USA) and cultured in DMEM supplemented with 10% FBS and antibiotics (100 U/mL penicillin and 100 U/mL streptomycin) at 37°C in a humidified 95% O_2_ and 5% CO_2_ atmosphere. The medium was replaced every day and the cells were passaged every 2–3 days to maintain logarithmic growth.

### Cell viability

The effect of SIN on the viability of RAW264.7 macrophages was determined using the cell counting kit-8 (CCK-8) according to the manufacturer's instructions. RAW264.7 cells were plated in 96-well plates at a density of 1 × 10^4^ cells per well. After incubation for 24 h, the medium was withdrawn. The cells were washed with PBS and separately incubated with different concentrations of LPS (0, 0.01, 0.05, 0.1, 0.5, 1, 2, 5, 10, and 20 μg/mL) or SIN (0, 0.1, 1, 5, 10, 25, 50, 100, 500, and 1,000 μg/mL) for 24 h. Next, to evaluate the protective function of SIN against LPS, RAW264.7 were pre-incubated with 50 μg/mL SIN for 0, 0.5, 1, 1.5, 2, 2.5, 3, 3.5, and 4 h and then with 50 μg/mL SIN and 1 μg/mL LPS (co-stimulation) for another 24 h. After a 2-h pre-incubation, RAW264.7 were pre-incubated with various concentrations of SIN (0, 1, 10, 50 μg/mL) for 2 h and then persistently incubated with SIN with or without 1 μg/mL LPS for an additional 24 h. To test the final cell viability, 10 μL WST-8 was added to each well and incubated for 2 h. The optical density was read at 450 nm using a multifunction microplate reader (Bio-Tek, Winooski, VT, USA).

### LPS-induced inflammation cell model

SIN was freshly prepared, dissolved to 5 mg/mL in a mixed solvent of phosphate-buffered saline (PBS) and DMSO (v/v = 9:1), and diluted to target concentration with DMEM before use. LPS (Sigma, St. Louis, MO) was dissolved in PBS to make a stock solution (5 mg/mL) by sonication for 2 min, after which aliquots were obtained and stored at −80°C until use. RAW264.7 cells were seeded and incubated at 37°C overnight. For the inflammatory cytokine array, qRT-PCR analysis and ELISA assessments, cells were pre-treated with either 50 or 100 μg/mL SIN in serum-free medium for 2 h and then persistently incubated for another 24 h with or without subsequent exposure to 1 μg/mL LPS.

### Cytokine array

Approximately 1.0 × 10^7^ cells/100 mm dish were seeded in a 6-well plate and treated as described above. After incubation at 37°C, serum-free medium was harvested from cells pre-treated with SIN (50 or 100 μg/mL) for 2 h, co-stimulated with LPS (1 μg/mL) for another 24 h. The serum-free medium was then centrifuged at 700 × *g* for 10 min to remove cell debris, and the supernatant was collected and stored at −80°C for cytokine array analysis. Cytokine array was performed using a Mouse Inflammation Antibody Array C1 (RayBiotech, Norcross, GA, USA, CODE: AAM-INF-1) capable of semi-quantitative detection of 40 mouse proteins, following the manufacture's protocol. ImageQuant LAS4000 Scanner (GE Healthcare Corporate) was used for the densitometry analysis of the array. The Scanner analysis tool for AAM-INF-1 (RayBiotech) was used to automatically analyze the antibody array data.

### Quantitative real-time polymerase chain reaction (qRT-PCR)

RNA was isolated from Raw264.7 using Trizol® reagent (Carlsbad, CA, USA) according to the manufacturer's protocol. Total RNA (1 μg/sample) was incubated with the components of the PrimeScript® RT reagent kit (Takara Bio, Shiga, Japan, Code No. RR036A) at 37°C for 15 min. The cDNA was then amplified with SYBR® *Premix Ex Taq*™ (Tli RNaseH Plus) Kit (Takara Bio, Shiga, Japan, Code No. RR420A) using ABI 7500 (Applied Biosystems, Foster City, CA). All operations followed the manufacturer's protocol. The mRNA expression of all genes was normalized to the housekeeping gene, β*-actin*. The fold changes between groups were calculated using the Ct value with the 2^−ΔΔCt^ method (ΔCt = Ct_target gene_ − Ct_β−*actin*_). Primers were designed according to published sequences (see Supplementary materials and methods; Table S1).

### Evaluation of anti-inflammatory effects of SIN on macrophages *in vitro*

Due to the specific proposed roles of macrophages in inflammatory and immune reactions, we tested the anti-inflammatory effect of SIN on the LPS-activated macrophage cell line, RAW 264.7. RAW 264.7 cells (1 × 10^7^ cells per well) were incubated with serum-free medium for 12 h in 6-well plates, pre-treated with SIN (10, 50 μg/mL) for 2 h, and co-stimulated with SIN and LPS (1 μg/ml) for another 24 h. We measured the concentration of selected cytokines, including IL-6, granulocyte-macrophage colony-stimulating factor (GM-CSF), IL-12 p40/P70, IL-1α, TNF-α, IL-1β, KC, Eotaxin-2, IL-10, M-CSF, RANTES, and MCP-1 in the culture supernatants by ELISA (see Supplementary materials and methods for kits used; Table S2).

### Enzyme linked immunosorbent assay (ELISA)

Blood samples were collected from all patients and volunteer subjects. All patients who participated in this study provided informed consent. Cytokine levels were assayed using ELISA kits (RayBiotech Norcross, GA, USA) (See Supplementary materials and methods; Table S2).

### Collagen-induced arthritis (CIA) mice model and SIN treatment

All animal procedures were conducted according to National Institutes of Health guidelines and approved by the Institutional Animal Care and Use Committee of Nanjing University of Chinese medicine. The collagen-induced arthritis mouse model was established as previously described (29). Briefly, male DBA/1 mice (6–8-weeks-old) were obtained from Beijing Vital River Laboratory Animal Technology Co., Ltd. (Charles River Laboratories department in Beijing, China), and kept under specific pathogen-free conditions. Chick collagen type II (CII) (Chondrex, Redmond, Washington, USA) was dissolved in 0.05 M acetic acid to a concentration of 2 mg/mL and emulsified with a complete Freund's adjuvant (CFA, 2 mg/mL *M. Tuberculosis* H-37 RA, Chondrex). At the beginning of the experiments (day 0), the mice were immunized with a 0.1 mL emulsion containing 100 μg of collagen at the tail base with a glass syringe and 25-G needles and then administered a booster (on day 21) with the same preparation of collagen and incomplete Freund's adjuvant (IFA, Chondrex). The mice were divided into four experimental groups: (1) Sham animals were maintained up to day 42. (2) vehicle-treated CIA mice received saline 0.1 mL i.g. (3) SIN-treated CIA mice received 50 mg/kg/day SIN i.g. (4) SIN-treated CIA mice received 100 mg/kg/day SIN i.g. for 20 days after the booster injection. Mice were sacrificed under anesthesia on day 42 after primary injection, and knee joints were isolated.

### Histomorphometric and histologic analyses

CIA was scored on a scale of 0–16 (0–4 for each paw, adding the scores for all 4 paws) using the following criteria: 0 score, normal paw; 1 score, one toe inflamed and swollen; 2 score, more than one toe, but not entire paw, inflamed and swollen or mild swelling of entire paw; 3 score, entire paw inflamed and swollen. 4 score, very inflamed and swollen paw or ankylosed paw. If the paw is ankylosed, the mouse cannot grip the wire at the top of the cage. Hind paws were fixed for 48 h in 10% buffered formalin and decalcified in 15% EDTA. The paws were then embedded in paraffin, and serial 5-μm sagittal sections of whole hind paws were cut and stained with hematoxylin and eosin. Two independent observers assessed the tissue for degree of synovitis by microscopic evaluation under blinded conditions. The evaluation criteria for inflammation severity and cartilage damage were described previously (30). Synovitis was graded on a scale of 0 (no inflammation) to 4 (severely inflamed joint) based on the extent of infiltration of inflammatory cells into the synovium. Sections were also stained with Safranin O–fast green to determine cartilage degradation. Safranin O staining was scored with a semi quantitative scoring system of 0 (no loss of proteoglycans) to 4 (complete loss of proteoglycans). The terminology and units were described according to international guidelines (31).

### Synovial tissue preparation and cell isolation from spleen and draining lymph nodes for flow cytometry analysis

Synovial tissue was prepared according to the protocol by Razawy et al. (32). The cell isolation procedures were performed partly by referring to previous studies (33, 34). Flow cytometric analyses were performed using Cytomics™ FC 500 (Beckman Coulter, CA. USA). For human blood samples and mice spleen, erythrocytes were lysed by a 10-min incubation with RBC Lysis Buffer (eBioscience) according to the manufacturer's protocol. PBMCs were washed well with PBS and resuspended in PBS with 1% fetal calf serum (Gibco). Synovial macrophages were identified as CD11b^+^F4/80^+^CD64^+^ (32, 35), spleen or draining lymph node monocytes/macrophages were identified as CD11b^+^Ly6C^+^CD43^+^ (36), and PBMC monocytes were identified as CD14^+^CD16^+^ (17, 37). + denotes an expression level that was ~10-fold above the isotype control or a cell whose subpopulation was separated from the others and could be obviously defined. FITC-CD11b (eBioscience) antibody and APC-F4/80, APC/Cy7-CD64, APC-Ly6C, APC/Cy7-CD43, FITC-CD14, and PE-CD16 antibodies (Biolegend) were used to detect these immune cell subsets. The following gating scheme was used: firstly, main population without cell debris (>90% cells) was defined by forward scatter (FSC) and side scatter (SCC). Then, the cells of this FSC/SSC gate were evaluated for expression of cell surface markers. For synovial macrophages, CD11b^+^F4/80^+^ subpopulation was identified through isotype control (>10-fold change vs. control). Next, CD11b^+^F4/80^+^CD64^+^ (Ly6C^−^) subpopulation was delineated in APC/Cy7-CD64 vs. APC-Ly6C plot. For monocytes/macrophages of spleen or draining lymph nodes, CD11b^+^ cells were gated by FITC-CD11b antibody and through isotype control (>90% cells vs. control). Subsequently, the CD11b^+^ Ly6C^+^CD43^+^ subpopulation was identified and delineated in APC-Ly6C vs. APC/Cy7-CD43 plot. For PBMC monocytes, CD14^+^CD16^+^ subpopulation was identified and delineated in FITC-CD14 vs. PE-CD16 plot. A 10-fold change of CD14^+^CD16^+^ cells over isotype control was observed. Each of the cell suspensions incubated without any antibody was set as an unstained control. The placebo group in the animal experiments and PBMCs of healthy donors were set as biological controls. The detailed antibody information and isotype control are listed in Supplementary materials and methods, Table S3.

### Patients, therapeutic regimen, response assessment

This study was conducted in Nanjing Hospital of Chinese Medicine, Nanjing, China from March 2016 to July 2017. Adults (≥18 years of age) with active RA (defined as DAS28-ESR ≥ 2.6) were eligible for enrollment. Forty-nine newly diagnosed RA patients met the 1987 American Rheumatism Association criteria for RA (38), without receiving prior hormone therapy and immunosuppressive or biologic therapies for at least 2 months; 20 matched healthy volunteers with no clinical symptoms of RA served as controls. All of the enrolled patients were early diagnosed RA patients (disease duration <2 years). A randomized, controlled trial was conducted to evaluate SIN clinical anti-inflammatory effects. Female patients in pregnancy or lactation periods and patients with asthma history were excluded from the treatment. Patients with other autoimmune inflammatory disorders, such as ankylosing spondylitis, psoriasis, multiple sclerosis, sarcoidosis, and inflammatory bowel diseases were excluded from the study. Regular examination of the liver and kidney function and blood routine examination were carried out during our observation. Individual therapy was discontinued if any adverse reaction occurred (e.g., dizziness, nausea, vomiting, skin rash, and abnormality in blood biochemical examination).

The selected 49 RA patients were randomly allocated to the experimental group (25 patients) and positive control group(24 patients); patients in each group received Zhengqing Fengtongning tablets (60–120 mg) orally 2 times a day and MTX (7.5–10 mg) and folic acid tablets (5 mg) orally once a week. Combination use of Diclofenac Sodium Sustained Release tablets (75 mg qn) was allowed if necessary. The period of treatment for each group was 3 months. Combination use of DMARDs, hormonal medicines, tripterygium tablets, and non-steroidal anti-inflammatory drugs (NSAIDs), which could affect the assessment of the disease therapy was forbidden during the course of treatment. However, the use of diclofenac sodium sustained release tablets was allowed except during the 7 days period before blood review. The study was approved by the Medical Ethics Committee of the Third Affiliated Hospital of Nanjing University of Chinese Medicine, Nanjing, China and adhered to the tenets of the Declaration of Helsinki (Permission No.: KY2015025, Date: 2015-3-23). All patients provided written informed consent before they were included in the study. Disease activity was assessed by the 28-joint disease activity score (DAS28) (39). DAS28 was evaluated by a rheumatologist who was blinded to the medications used or laboratory test results. The patients were categorized into good, moderate, or non-responders based on the amount of change in DAS28 and the attained level of DAS28. Good responders were defined as patients who had a decrease in DAS28 from baseline (ΔDAS28) of >1.2 and a DAS28 of <3.2 at week 12; moderate responders had either ΔDAS28 of >1.2 and a DAS28 of ≧3.2 at week 12 or ΔDAS28 of 0.6–1.2 and a DAS28 of <5.1 at week 12; and non-responders had either ΔDAS28 of <0.6 or a DAS28 of ≧5.1 at week 12 (40).

### Medicines and chemical reagent for patients

Zhengqing Fengtongning Retard tablets (containing 20 mg/tablet SIN) were obtained from Hunan Zhengqing Pharmaceutical Group Ltd Company (Hunan, China; Z20010174), methotrexate tablets were provided by Shanghai Xinyi Pharmaceutical Co., Ltd (Shanghai, China; H31020644), and folic acid tablets were obtained from Changzhou Pharmaceutical Factory Co., Ltd (Changzhou, China; H32023302). Diclofenac sodium sustained-release tablets were provided by Beijing Novartis Pharma Ltd (Beijing, China; H10980297).

### Statistical analysis

Data were expressed as means ± SD. Comparison of the difference of means was performed by one-way ANOVA using SPSS 17.0 software. For all quantitative data, statistical analyses were performed using GraphPad Prism 7.0 software. If ANOVA indicated a significant difference, Bonferroni *post-hoc* test was performed to assess the difference between groups. Paired sample *t*-test was applied to compare serum levels of cytokines during follow-up for the RA patients before and after SIN or MTX therapy. Chi square test or Fisher exact test was used to compare the rates of response. The correlation coefficient was obtained by the non-parametric Spearman's rank correlation test. The Wilcoxon paired signed rank test was used to compare clinical score and the percentages of each myeloid cell or monocyte subset. *P* < 0.05 was considered statistically significant.

## Results

### SIN prevents lipopolysaccharide (LPS)-induced RAW267.7 macrophage damage

To evaluate the *in vitro* anti-inflammatory effects of SIN based on concentration and detect its cytotoxic potential, cell viability assay of SIN-RAW264.7 cells was performed. CCK-8 assays indicated that the cells were stable after incubation with 0.1–50 μg/mL SIN for 24 h, suggesting that the cells were viable, whereas higher concentrations (100–1,000 μg/mL) significantly reduced cell viability (Figure 1B). Therefore, SIN concentrations of 10 and 50 μg/mL were selected for subsequent experiments. Besides, more than 1 μg/mL LPS could significantly reduce the cell viability (*P* < 0.05; Figure 1C). To obtain enough cells for cytokine evaluation, 1 μg/mL LPS was selected for further stimulation in the cell model. To test the proper pre-incubation time for 50 μg/mL SIN, we pre-treated RAW264.7 macrophages with 50 μg/mL SIN for 0–4 h, following co-LPS-activation (1 μg/mL) for an additional 24 h; 0 h data indicate that SIN and LPS were added to the cells simultaneously for 24 h. The result suggested that simultaneous addition of SIN and LPS to the cells did not significantly increase the cell viability. However, when the cells were pre-incubated with SIN for 1–2 h before LPS stimulation, cell viability was rescued and reached the peak at 2 h. In contrast, when the pre-incubation time was more than 2 h, the cell viability decreased significantly (Figure 1D, *P* < 0.05). These data indicated that 2 h was the appropriate pre-treatment time for rescuing LPS-suppressed cell viability. In addition, to test other SIN concentrations, RAW264.7 macrophages were pre-treated with SIN (1, 10, and 50 μg/mL) for 2 h and then continuously co-incubated with 1 μg/mL LPS for another 24 h. The results demonstrated that SIN pre-treatment and continued co-incubation with LPS increased the cell viability in a concentration-dependent manner (Figure 1E), indicating that SIN could protect RAW264.7 cells from LPS-induced damage but required an appropriate concentration and pre-incubation time. This may be due to the fact that LPS is a robust inflammatory inducer and thus, activate cells more quickly. Taken together, it can be concluded that SIN anti-inflammatory effects will be stronger under pre-incubation conditions. These experiments helped us to determine the *in vitro* experimental conditions.

### Screening data of cytokines induced by LPS or by co-incubation with SIN in RAW264.7

**Figure 2:**
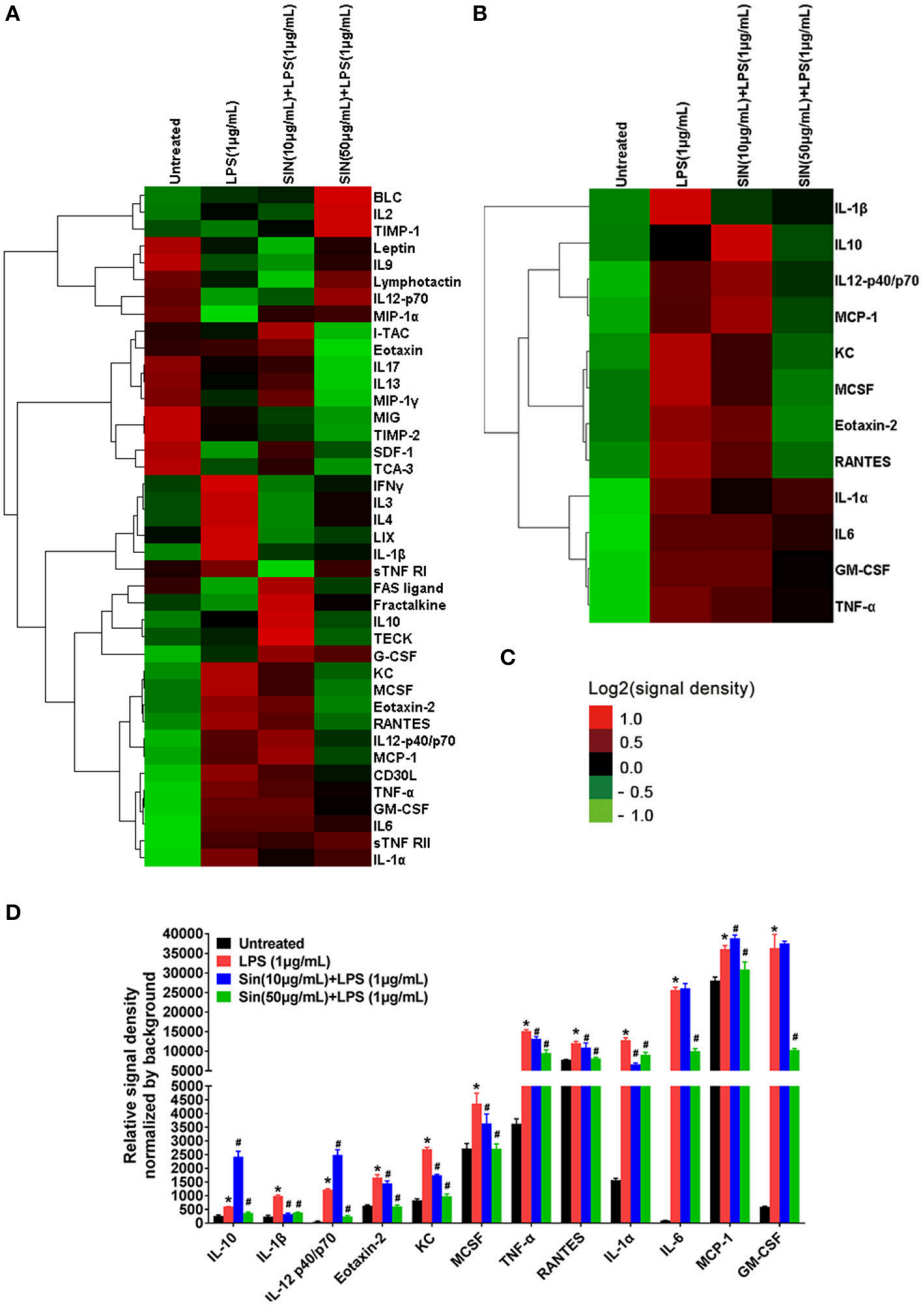
Hierarchical cluster of cytokines in the inflammatory array and the 12 cytokines which were significantly regulated by SIN (at least one comparison *P* < 0.05, 10 μg/mL SIN + LPS vs. LPS or 50 μg/mL SIN + LPS vs. LPS). **(A)** Hierarchical cluster of the 40 cytokines. **(B)** Hierarchical cluster of the 12 selected secreted cytokines (IL-6, GM-CSF, IL-12 p40, IL-1α, TNF-α, IL-1β, KC, Eotaxin-2, IL-10, M-CSF, RANTES, and MCP-1). **(C)** Color bar of the cluster analysis. Red, upregulated; green, downregulated. All the signal density values of cytokines were transformed to Log_2_ (signal density) and subtracted the density value-wise mean from the values of each cytokine, so that the mean value of each group was 0. Multiply all values in each group of data by scale factor S, so that the sum of the squares of the values in each row is 1.0. Cluster type, average linkage clustering. Figures were generated by Cluster 3.0 (Stanford University) and TreeView (Alok, version: 1.1.6r4). **(D)** Detailed relative signal density normalized by background of 12 selected cytokines. (*P < 0.05 vs. untreated; ^#^*P* < 0.05 vs. LPS treated RAW264.7).

Based on the experimental conditions obtained from the cell viability assay, we used inflammatory cytokine array to screen 40 cytokines induced by LPS in RAW264.7 cells and evaluated the SIN pre-incubation effects on the LPS-induced group. The cluster analysis of all cytokines is shown in Figure 2A. The detailed screening data of all the inflammatory cytokines secreted in each group are shown in Table S4. Screening and validation followed the workflow shown in Figure S1. The data showed that 11 cytokines were upregulated (Fold change >1.5, *P* < 0.001) and seven were downregulated (Fold change < 1, *P* < 0.05) in the LPS group compared with those in the untreated group. For the 10 μg/mL SIN pre-incubation plus 1 μg/mL LPS group, we found that 12 cytokines were upregulated (Fold change >1.0, *P* < 0.05) and nine were downregulated (Fold change < 1.0, *P* < 0.05) compared with the single LPS induced group, which included three upregulated cytokines (Fold change >1.5, *P* < 0.05) and one downregulated cytokine (Fold change < 0.5, *P* < 0.05). However, unlike 50 μg/mL SIN, 10 μg/mL SIN could not attenuate most of the secreted cytokines. In 50 μg/mL SIN plus 1 μg/mL LPS group, at least six cytokines were significantly downregulated compared with those in the single LPS induced group (Fold change < 0.5, *P* < 0.01). The 12 cytokines, which were significantly induced by LPS and could be remarkably reversed by SIN (at least one comparison *P* < 0.05) are listed in Table 1. Cluster analysis was performed with the 12 cytokines as shown in Figure 2B, followed by validation using ELISA. Obviously, because the abundance and SIN regulation degree of these cytokines differ in the medium, the 12 cytokines were distributed into clusters. For example, one of the clusters, IL-10, IL-12-p40/p70, MCP-1, KC, MCSF, Eotaxin-2, and RANTES, could be closer to the untreated group level under 50 μg/mL SIN treatment, and the other cluster, IL-1α,IL6,GM-CSF and TNF-α clustered at the middle level compared with the 1 μg/mL LPS group. As shown in Figure 2B, 50 μg/mL SIN exhibited a higher cytokines-mediating effect than 10 μg/mL, except IL-1β, which could be significantly downregulated by 10 μg/mL SIN. The color bar of the cluster analysis is shown in Figure 2C. The relative signal density of the 12 cytokines is shown in Figure 2D. The original scan images of each sample and histograms of all data are shown in Figure S2.

**Table 1:** LPS-induced and SIN reversed target cytokines in inflammatory cytokine array (*n* = 4, *P* < 0.05).

**Cytokines**	**LPS/Untreated**		**SIN (10** μ**g/mL)** + **LPS (1** μ**g/mL)/LPS (1** μ**g/mL)**		**SIN (50** μ**g/mL)** + **LPS (1** μ**g/mL)/LPS (1** μ**g/mL)**	
	**Fold change**	***P*-value**	**Fold change**	***P*-value**	**Fold change**	***P-*value**
IL-6	265.9496	0.0000	1.0177	0.5491	0.3915	0.0000
GM-CSF	61.0811	0.0000	1.0333	0.5213	0.2836	0.0000
IL-12 p40	25.1822	0.0000	2.0361	0.0000	0.2003	0.0000
IL-1α	8.168	0.0000	0.5181	0.0000	0.710	0.0000
TNF-α	4.1769	0.0000	0.8731	0.0008	0.6314	0.0000
IL-1β	4.0747	0.0000	0.3337	0.0000	0.3891	0.0000
KC	3.2293	0.0000	0.6482	0.0000	0.3643	0.0000
Eotaxin-2	2.6095	0.0000	0.8714	0.0205	0.3660	0.0000
IL-10	2.3112	0.0000	3.9922	0.0000	0.6157	0.0000
M-CSF	1.6015	0.0002	0.8336	0.0297	0.6221	0.0002
RANTES	1.5425	0.0000	0.9057	0.118	0.6773	0.0000
MCP-1	1.2874	0.0000	1.0760	0.0036	0.8555	0.0025

### SIN reverses secretion and mRNA expression levels of inflammatory cytokines in LPS-induced RAW264.7 macrophages

**Figure 3:**
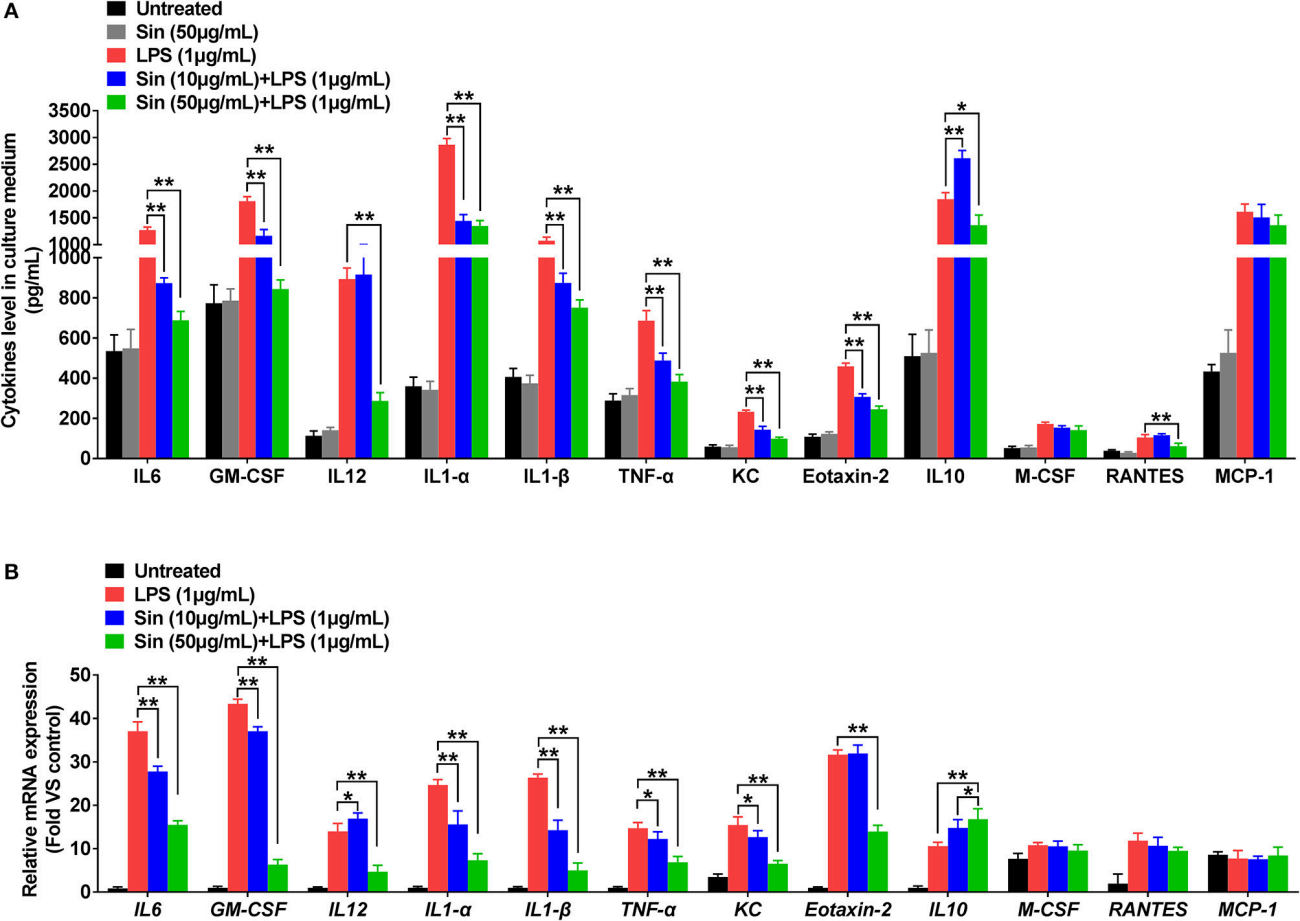
RAW 264.7 cells were incubated with serum-free medium for 12 h in 6-well plates, pre-treated with SIN (10 or 50 μg/mL) for 2 h, and finally co-stimulated with LPS (1 μg/mL) for another 24 h. **(A)** IL-6, GM-CSF, IL-12 p40, IL-1α, TNF-α, IL-1β, KC, Eotaxin-2, IL-10, M-CSF, RANTES, and MCP-1 release from conditioned medium was measured by ELISA, respectively. The values represent the means ± SD of triplicate experiments (*P < 0.05, **P < 0.01). **(B)** Relative mRNA levels of the 12 selected cytokines by arrays were determined using RT-PCR. The results of RT-PCR were normalized to β-actin and expressed as fold change to the control. The values represent the means ± SD of triplicate experiments. *P < 0.05 vs. LPS treated RAW264.7.

The cytokine array data was further confirmed by ELISA to assess the role of the mouse macrophage cell line RAW264.7 in response to LPS stimulation. The ELISA results confirmed that 1 μg/mL LPS significantly increased IL-6, GM-CSF, IL-12 p40, IL-1α, TNF-α, IL-1β, KC, Eotaxin-2, IL-10, M-CSF, RANTES, and MCP-1 levels in RAW264.7 cells compared to those in normal cells (*n* = 3, *P* < 0.05). In the presence of LPS stimulation, SIN showed a concentration-dependent reduction in secretion of IL-6, GM-CSF, IL-1α, IL-1β, TNF-α, KC, and Eotaxin-2 compared with those in RAW264.7 cells treated with LPS alone. Contrarily, IL-10, which served as an anti-inflammatory cytokine, was rescued by 10 or 50 μg/mL SIN under LPS treatment (Figure 3A, ^*^P < 0.05, ^**^P < 0.01). Obviously, the data of all the kits showed no significant difference between RAW264.7 treated with 50 μg/mL SIN-alone group and the blank untreated group (*P* > 0.05), consistent with the data of 50 μg/mL SIN (Figure 1B). Specifically, ELISA validation was partly inconsistent with the cytokine array data. For example, LPS-stimulated IL-12 increase was not attenuated by 10 μg/mL SIN. IL-10 secretion was increased under 10 μg/mL SIN treatment but was suppressed under 50 μg/mL SIN treatment compared with LPS induced treatment alone. In addition, 10 μg/mL SIN could not attenuate RANTES secretion induced by LPS, but 50 μg/mL SIN worked well (^**^P < 0.01). M-CSF and MCP-1 did not show any significant change under 10 or 50 μg/mL SIN treatment (*P* > 0.05), although their secretions were slightly decreased by 10 or 50 μg/mL SIN under LPS stimulation.

The inflammatory cytokines generated in the cytoplasm and secreted by macrophages. To detect the cytokines generated in the cytoplasm, we further examined whether SIN inhibited LPS-induced mRNA expression levels of the 40 inflammatory cytokines aforementioned using qRT-PCR (Figure S3). As shown in Figure 3B, 50 μg/mL SIN treatment significantly suppressed LPS-induced mRNA overexpression of IL-6 (*P* < 0.0001), GM-CSF (*P* < 0.0001), IL-12 (*P* = 0.0024), IL-1α (*P* < 0.0001), IL-1β (*P* < 0.0001), TNF-α (*P* = 0.0018), KC (*P* = 0.0015), and Eotaxin-2 (*P* = 0.0034). Moreover, 10 μg/mL SIN also significantly downregulated the expression of these cytokines (^*^P < 0.05, ^**^P < 0.01) except Eotaxin-2 (*P* > 0.05). This indicated that transcription of the mRNA of these six cytokines could be more sensitively regulated by SIN. Particularly, the expression of the conventional anti-inflammatory cytokine, IL-10 (*P* = 0.0139), was upregulated by 50 μg/mL SIN compared to 1 μg/mL LPS-stimulation group. In addition to these cytokines, LPS-induced M-CSF and RANTES expression could not be suppressed by 10 μg/mL SIN or 50 μg/mL SIN. Moreover, there was no change in MCP-1 mRNA expression under SIN treatment or LPS stimulation.

These results were confirmed by the results of ELISA and qRT-PCR. However, further *in vivo* studies are needed to confirm SIN anti-inflammatory effects, especially the negative results about M-CSF, RANTES, and MCP-1.

### SIN rescues inflammation and cartilage damage in collagen-induced arthritis DBA/1 mice

**Figure 4:**
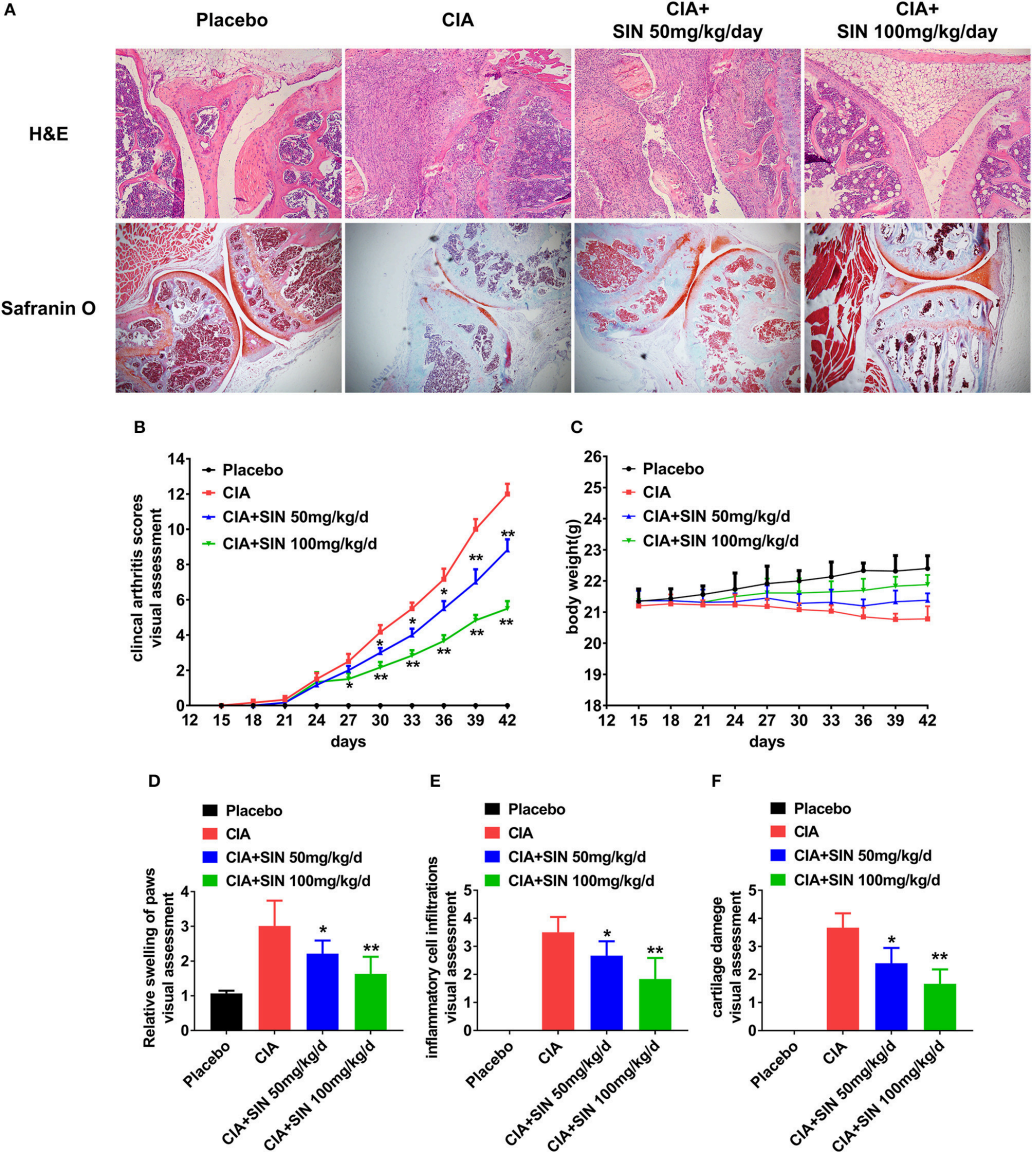
SIN rescues inflammation and cartilage damage in collagen-induced arthritis DBA/1 mice. **(A)** H&E staining and Safranin-O staining of the ankle joints of each group. **(B)** Clinical arthritis score was evaluated every 3 days in each group. From day 30 after immunization or the first time, SIN could ameliorate clinical arthritis scores of CIA. The values represent the means ± SD (*n* = 6). Significance was analyzed by Wilcoxon rank-sum test (*P < 0.05, **P < 0.01). **(C)** Body weight from each group had no significant difference. However, SIN slightly led to body weight gain in CIA mice. The values represent the means ± SD (*n* = 6). Significance was analyzed by unpaired 2-sided *t*-test. **(D)** Swelling of paws, **(E)** inflammatory cell infiltrations, **(F)** cartilage damage could also be attenuated by SIN. Data are presented as the mean ± SD (*n* = 6). Significance was analyzed by Wilcoxon rank-sum test (*P < 0.05, **P < 0.01).

To further investigate SIN anti-inflammatory effects on RA *in vivo*, we evaluated its therapeutic effects in CIA DBA/1 mice. As shown in Figure 4A, histological analysis was performed on the ankle joints of placebo-treated and SIN-treated CIA mice. H&E staining revealed that the joints of mice treated with 50 or 100 mg/kg SIN daily demonstrated less inflammatory cell infiltration and synovial hyperplasia. In addition, selected joint sections were stained with Safranin O to evaluate calcium content in the articular cartilage. The CIA mice had reduced Safranin-O staining, indicating diminished calcium content in osteoblast. In contrast, the decreased Safranin-O staining was rescued in the CIA animals administered with SIN. Furthermore, SIN (50 or 100 mg/kg) significantly ameliorated clinical arthritis scores of CIA confirmed by visual inspection (Figure 4B). There were no differences in body weight changes between the groups, but SIN slightly reversed body weight loss induced by CII (Figure 4C). Quantitative histological analysis revealed that the swelling paws score (Figure 4D), inflammation score (Figure 4E), and cartilage damage score (Figure 4F) were significantly decreased in SIN-treated CIA mice compared to placebo-treated mice (^*^P < 0.05, ^**^P < 0.01). These results suggest that SIN has the potential to ameliorate the development of CIA.

### SIN suppresses inflammatory cytokine secretion in mice with CIA

**Figure 5:**
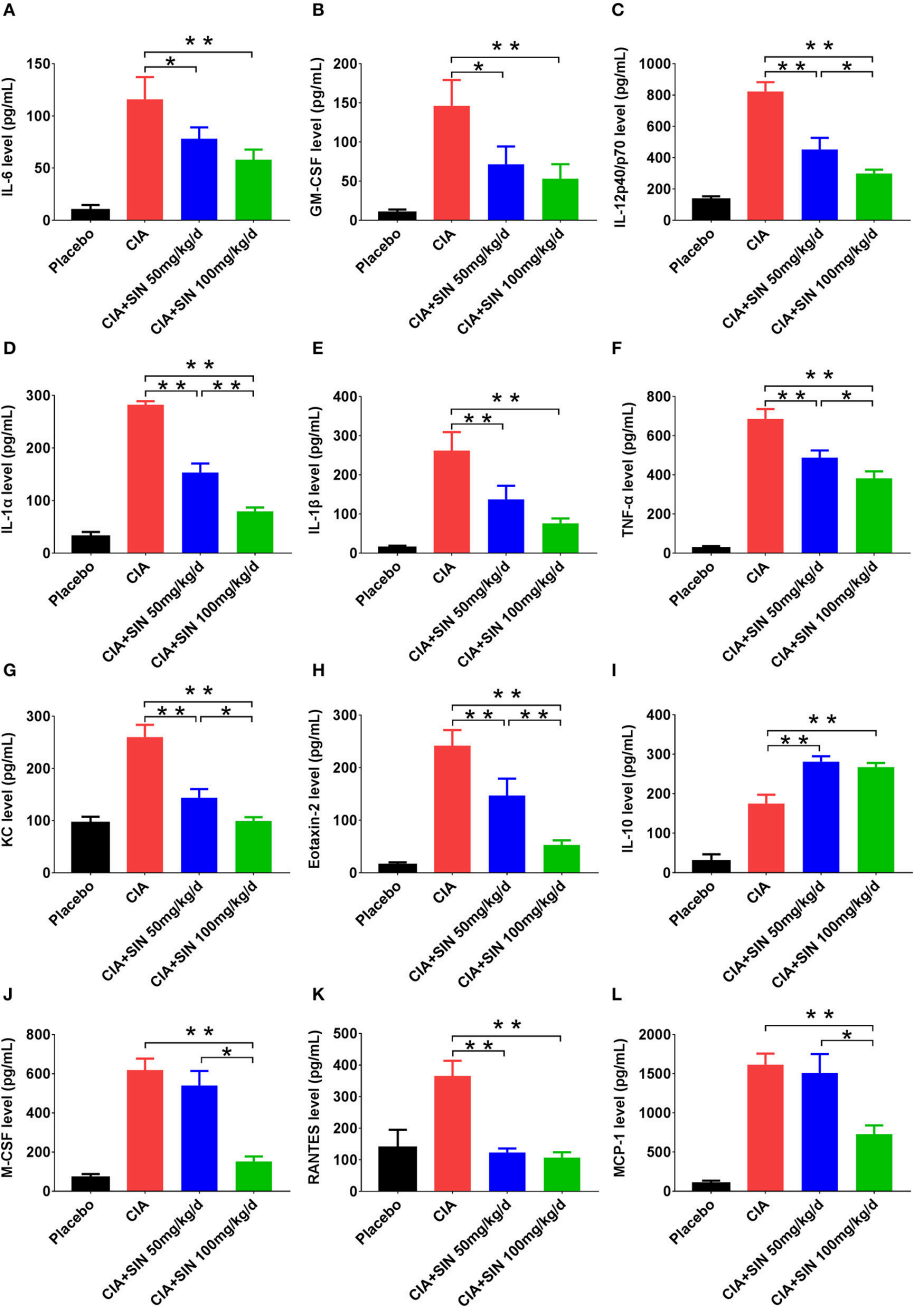
Cytokines screened from LPS-induced RAW264.7 and SIN treatment *in vitro* were validated *in vivo*. Serum levels of **(A)** IL-6, **(B)** GM-CSF, **(C)** IL12-p40/p70, **(D)** IL-1α, **(E)** IL-1β, **(F)** TNF-α, **(G)** KC, **(H)** Eotaxin-2, **(I)** IL-10, **(J)** MCSF, **(K)** RANTES, **(L)** MCP-1 from mice in each group were measured by ELISA, respectively. Data are presented as the mean ± SD (*n* = 6). Significance was analyzed by unpaired two-sided *t*-test (*P < 0.05, **P < 0.01).

The secretion of 12 inflammatory cytokines was first validated *in vitro*, and further validated *in vivo* by ELISA. The secretion of all 12 cytokines was increased in serum of CIA mice compared to that in placebo-treated mice. IL-6, GM-CSF, IL-12 p40, IL-1α, IL-1β, TNF-α, KC, and Eotaxin-2 secretion in CIA mice was suppressed by 50 or 100 mg/kg SIN daily administration in a concentration-dependent manner (Figures 5A–H). However, IL-10 secretion was increased under 50 or 100 mg/kg/day SIN treatment (Figure 5I). For M-CSF and MCP-1, only 100 mg/kg/day SIN, but not 50 mg/kg/day could inhibit their secretion (Figures 5J,L). In addition, 50 mg/kg/day and 100 mg/kg/day significantly attenuated RANTES secretion, but there was no difference between the two in terms of their secretion (Figure 5K).

### SIN reduces the percentage of CD11b^+^F4/80^+^CD64^+^ synovial macrophages and CD11b^+^Ly6C^+^CD43^+^ monocytes/macrophages in CIA mice

**Figure 6:**
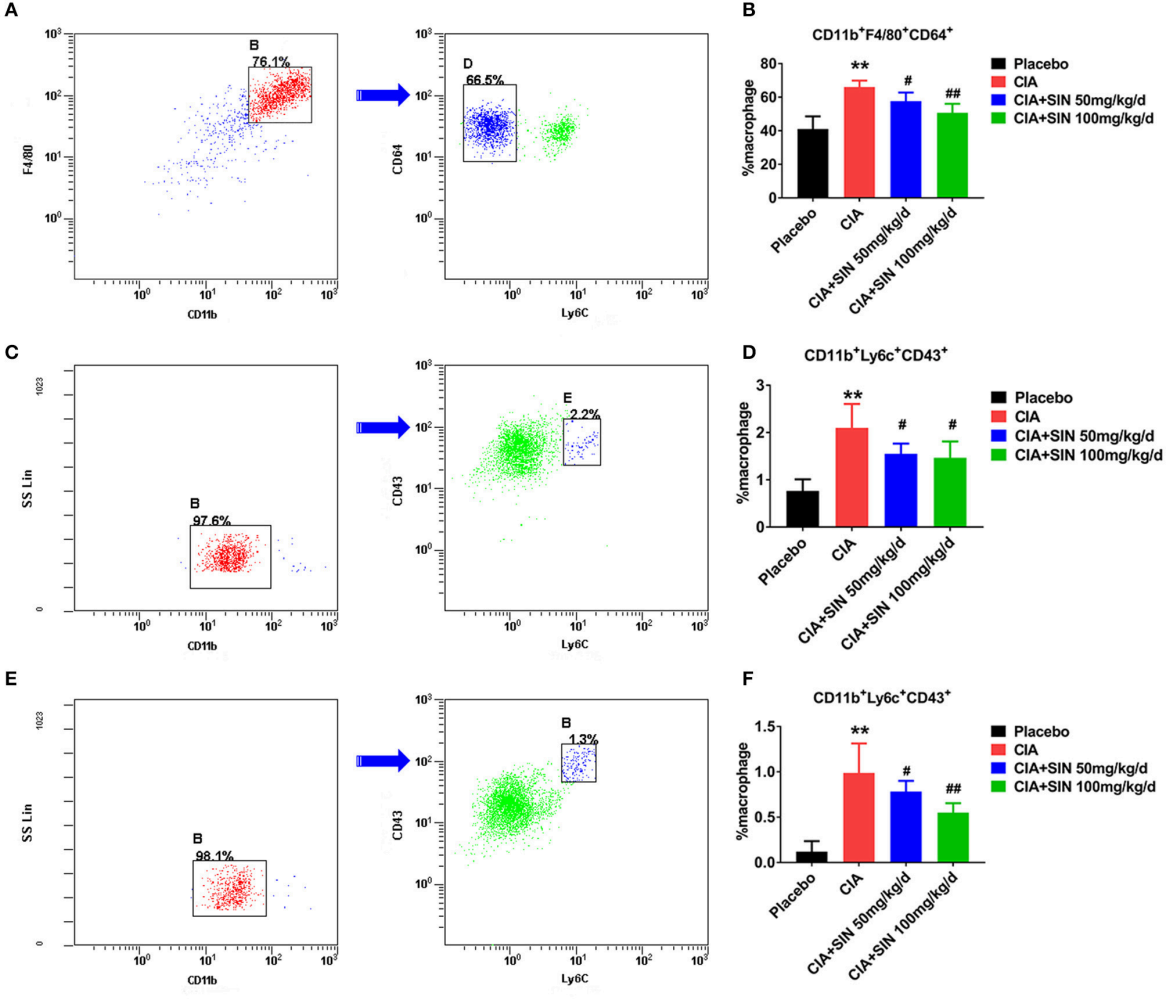
SIN reduces the percentage of CD11b^+^F4/80^+^CD64^+^ synovial macrophages and CD11b^+^Ly6C^+^CD43^+^ monocytes/macrophages in CIA mice. **(A)** A representative flow cytometry analysis of ankle macrophages in CIA mice under steady state conditions presenting the gating strategy. After exclusion of doublets and debris, and gating out granulocytes, dendritic cells, and B cells, synovial macrophages were identified as CD11b^+^F4/80^+^CD64^+^. After removing Ly6C^+^ cells, synovial tissue resident macrophages were identified as CD11b^+^F4/80^+^ CD64^+^ (Ly6C^−^). Arrow denotes a parent population being displayed in a subsequent plot. Cells positive for CD11b and F4/80 are gated and displayed in a plot of CD64 vs. Ly6C, in which the CD11b^+^F4/80^+^CD64^+^ cell population is present in left delineation gate. **(B)** Analysis of the total synovial macrophages (CD11b^+^F4/80^+^) and the percentage of synovial tissue resident macrophages from ankles of placebo-treated, CIA, CIA plus 50 or 100 mg/kg/d SIN treated mice. **(C)** Spleen cells were depleted of T, B, and red blood cells and stained with antibodies to CD11b, CD43, and Ly6C. Spleen monocytes/macrophages were identified as CD11b^+^Ly6C^+^CD43^+^. CD11b^+^ cells were initially gated on the basis of a monocyte/macrophage phenotype. Gating strategies were based on fluorescence minus one control, and numbers in gates represent % specific binding. A representative gating result about CIA mice is shown. Arrow denotes a parent population being displayed in a subsequent plot. Cells positive for CD11b is gated and displayed in a plot of CD43 vs. Ly6C, in which the CD11b^+^Ly6C^+^CD43^+^ cell population is present in right delineation gate. **(D)** Analysis of the percentage of CD11b^+^Ly6C^+^CD43^+^ monocytes/macrophages from spleens of placebo-treated, CIA, CIA plus 50 or 100 mg/kg/d SIN treated mice. **(E)** Draining lymph nodes (inguinal and popliteal) were isolated and prepared for staining CD11b, CD43, and Ly6C. CD11b^+^Ly6C^+^CD43^+^ monocytes/macrophages were identified in the same manner as those of the spleen. A representative gating result about CIA mice is shown. Arrow denotes a parent population being displayed in a subsequent plot. Cells positive for CD11b is gated and displayed in a plot of CD43 vs. Ly6C, in which the CD11b^+^Ly6C^+^CD43^+^ cell population is present in right delineation gate. **(F)** Analysis of the percentage of CD11b^+^Ly6C^+^CD43^+^ monocytes/macrophages from draining lymph nodes (inguinal and popliteal) of placebo-treated, CIA, CIA plus 50 or 100 mg/kg/d SIN treated mice. Data are presented as the mean ± SD (*n* = 6). Significance was analyzed by unpaired 2-sided *t*-test (**P < 0.01 vs. Placebo; ^#^*P* < 0.05, ^##^*P* < 0.01 vs. CIA.).

To explain the anti-inflammatory mechanism of SIN, we detected two kinds of monocytes/macrophages in the CIA mouse model and under SIN treatment. Synovial macrophages were defined as CD11b^+^F4/80^+^CD64^+^(Ly6C^−^) and spleen or draining lymph nodes macrophages were defined as CD11b^+^Ly6C^+^CD43^+^. Gating scheme for synovial macrophages is shown in Figure 6A. Gating scheme for macrophage of spleen or draining lymph nodes is shown in Figures 6C,E. As shown in Figures 6A,B, the percentage of CD11b^+^F4/80^+^CD64^+^ macrophages in synovium was decreased by SIN from 66.1 ± 3.768 to 50.7 ± 5.326(100 mg/kg/day) in CIA mice (*n* = 6, *P* < 0.01). Analogously, SIN inhibited CD11b^+^Ly6C^+^CD43^+^ macrophage percentage in the spleen (Figures 6C,D) and draining lymph nodes (Figures 6E,F). The FACS analysis of synovial, spleen, and ankle macrophage subpopulations in 50 μg/mL SIN group is shown in Figure S4. The reduction in the number of these macrophages leads to the suppression of the secretion of inflammatory cytokines in CIA mice.

### SIN treatment significantly lessens disease activity and alleviates RA-specific clinical indicators in some but not all RA patients

Forty-nine early-diagnosed RA patients were divided into two groups and treated with two therapeutic regimens, SIN or MTX, as described above. The clinical characteristics, physiological indicators, and ΔDAS28 (DAS28_after_ − DAS28_before_) were compared before and after treatment following a 12-weeks course. As shown in Figure S5, under SIN treatment, the DAS28 of 25 RA patients fell from 4.19 (range: 2.76–6.27) before treatment to 3.28 (range: 1.54–5.9) after treatment (*P* < 0.01, Figure S5A). Under MTX treatment, the DAS28 of 24 RA patients fell from 4.44 (range: 2.82–6.8) before treatment to 2.88 (range: 1.53–5.16) after treatment (*P* < 0.001, Figure S5B). Consistently, RA-specific clinical indicators, including RF, CCP, ESR, and CRP were significantly reduced after treatment both in the SIN and MTX groups (Table 2). In the SIN group, 8 (32%) patients achieved good response, 9 (36%) patients achieved moderate response, and 8 (32%) patients had no response; while in the MTX group, 10 (41.7%) patients achieved good response, 11(45.8%) patients achieved moderate response, and 3 (12.5%) patients had no response (Table 3). There was no significant difference in remission rate between SIN and MTX treatments in patients with early diagnosed RA. These data indicated that SIN was an effective drug for RA remission, even though its remission rate was slightly lower than that of MTX.

**Table 2:** Baseline characteristics of the 20 healthy donors and clinical, biological and physiological indicators and therapeutic response after before and after treatment in RA patients.

**Groups**	**Healthy donors (*n* = 20)**	**Case (PRE-12 weeks)**	**Case (AFT-12 weeks)**	**Case (PRE-12 weeks)**	**Case (AFT-12 weeks)**
		**SIN (*****n*** = **25)**		**MTX (*****n*** = **24)**	
Age, years	53.65 ± 11.44	56.55 ± 13.92		57.84 ± 12.68	
Sex, no. (female %)	20 (70%)	25 (76%)		24 (70.83%)	
TJC28	–	9.33 ± 4.57	6.56 ± 3.48^*^	10.62 ± 4.79	5.81 ± 3.32^*^
SJC28	–	7.52 ± 3.81	4.57 ± 1.33^*^	7.25 ± 4.36	4.05 ± 1.62^*^
100-mmVAS	–	44.61 ± 13.74	31.53 ± 10.52^*^	45.83 ± 12.61	28.47 ± 8.28^*^
ESR, mm/h	5.56 ± 3.46	38.98 ± 9.49	23.74 ± 7.45^*^	41.02 ± 9.02^*^	20.51 ± 8.11^*^
CRP, mg/L	3.36 ± 1.73	21.84 ± 6.22	12.76 ± 3.93^*^	21.23 ± 6.80	13.82 ± 4.83^*^
RF (Positive %)	–	76%	32%^*^	75%	29.2%^*^
CCP (Positive %)	–	72%	36%^*^	79.2%	25%^*^
DAS28-ESR	–	4.34 ± 1.01	2.65 ± 0.71^*^	4.63 ± 1.08	2.44 ± 0.56^*^

*TJC, tender joint count; SJC, swollen joint count; RF, rheumatoid factor; CCP, cyclic citrullinated peptide antibody; DAS28, disease activity score of 28 joints; ESR, erythrocyte sedimentation rate; CRP, C-reactive protein*.

* *P < 0.05 vs. before treatment in the same patient group*.

**Table 3:** Numbers of patients responding to different treatment options.

**Treatment**	**Good responders ΔDAS28 > 1.2 and DAS28 < 3.2**	**Moderate responders 0.6 ≤ ΔDAS28 ≤ 1.2 and DAS28 < 5.1 or ΔDAS28 > 1.2 and DAS28 ≥ 3.2**	**Non-responders ΔDAS28 < 0.6 or DAS28 ≥5.1**	**Total**
SIN	8 (32%)	9 (36%)	8 (32%)	25 (100%)
MTX	10 (41.7%)	11 (45.8%)	3 (12.5%)	24 (100%)

*Fisher's exact test P = 0.328*.

### SIN modulates the secretion of inflammatory cytokines in the plasma of RA patients

**Figure 7:**
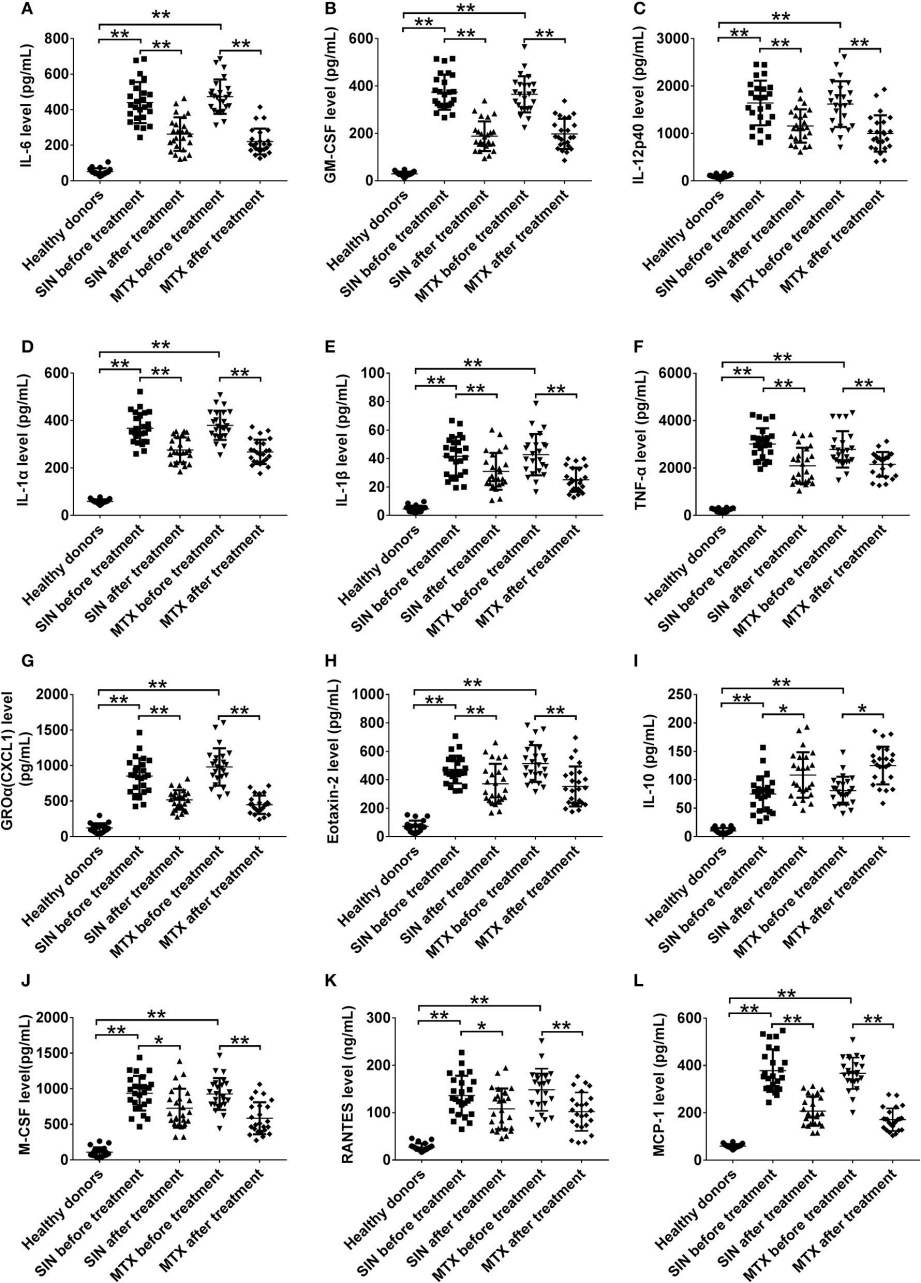
SIN modulates the secretory levels of inflammatory cytokines in the plasma of RA patients. **(A)** IL-6, **(B)** GM-CSF, **(C)** IL12-p40, **(D)** IL-1α, **(E)** IL-1β, **(F)** TNF-α, **(G)** GROα(CXCL1), **(H)**Eotaxin-2, **(I)** IL-10, **(J)** MCSF, **(K)** RANTES, and **(L)** MCP-1 secretion was detected by ELISA in heathy donors, before and after SIN and MTX treatments, respectively. Each dot represents one sample. Significance was analyzed by unpaired two-sided *t*-test (*P < 0.05, **P < 0.01).

**Figure 8:**
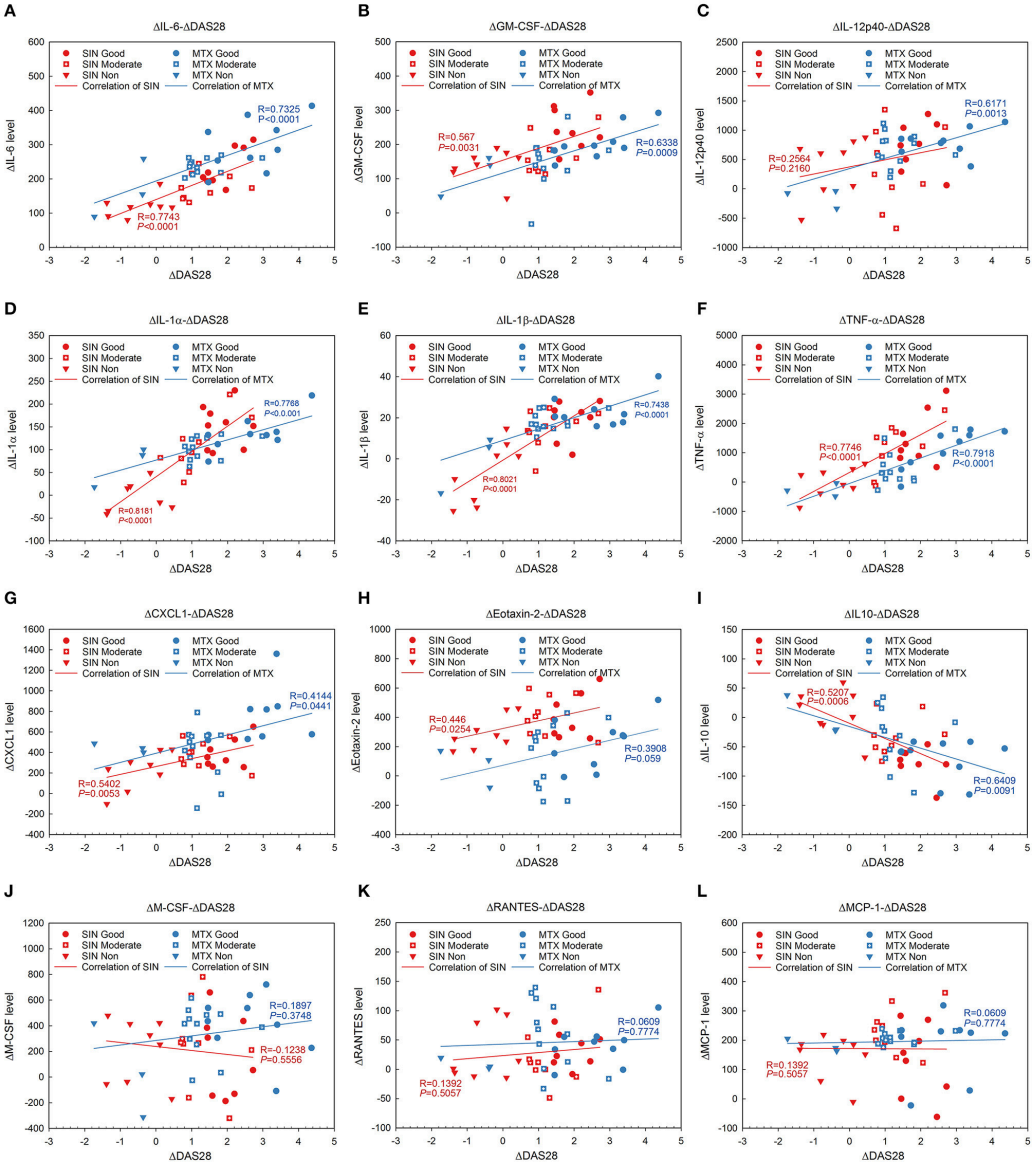
Linear regression and coloration analysis between cytokine changes and ΔDAS28 in RA patients before and after treated with SIN or MTX. **(A)** IL-6, **(B)** GM-CSF, **(C)** IL12-p40, **(D)** IL-1α, **(E)** IL-1β, **(F)** TNF-α, **(G)** CXCL1, **(H)** Eotaxin-2, **(I)** IL-10, **(J)** MCSF, **(K)** RANTES, **(L)** MCP-1. Positively correlated with ΔDAS28: IL-6, GM-CSF, IL-12 p40 (SIN *P* = 0.216, MTX *P* = 0.0013), IL-1α, IL-1β, TNF-α, CXCL1 (GROα), Eotaxin-2 (SIN *P* = 0.0254, MTX *P* = 0.059); negatively correlated with ΔDAS28: IL-10; no correlation with ΔDAS28: M-CSF, RANTES, and MCP-1. r, Pearson correlation coefficient. Red, SIN therapy; blue, MTX therapy. Round dot, good responders; square with white cross, moderate responders; inverted triangle, non-responders. Red line, correlation of SIN; blue line, correlation of MTX.

It was no doubt that at least 12 cytokines were modulated by SIN according to our data *in vivo* and *in vitro*. To explore the function of SIN in the clinical setting, the levels of these inflammatory cytokines were also determined before and after 12 weeks of treatment with SIN or MTX in RA patients. As shown in Figure 7, all of these cytokines in RA patients were upregulated dramatically than those in healthy donors. IL-6, GM-CSF, IL-12 p40, IL-1α, IL-1β, TNF-α, GROα, Eotaxin-2, M-CSF, RANTES, and MCP-1 secretion in RA patients were suppressed under SIN or MTX treatment (Figures 7A–H,J–L). IL-10 secretion was slightly upregulated by SIN or MTX (Figure 7I). There was no significant difference in controlling the secretion of these cytokines between SIN and MTX, which was consistent with the clinical response rate in Table 3. These results demonstrated that these 12 inflammatory cytokines could act as good indicators of RA occurrence. For early diagnosed RA patients, SIN was the drug of choice for inhibiting inflammation. In addition, correlation between ΔDAS28 and cytokine level changes before and after treatment was determined. Delta IL-6, GM-CSF, IL-12 p40, IL-1α, IL-1β, TNF-α, CXCL1 (GROα), and Eotaxin-2 secretion was positively correlated with ΔDAS28 (Figures 8A–H, R < 0, SIN or MTX *P* < 0.05). On the contrary, delta IL-10 was negatively correlated with ΔDAS28 (Figure 8I, R < 0, SIN and MTX *P* < 0.05). Delta M-CSF, RANTES, and MCP-1 had no correlation with ΔDAS28 (Figures 8J–L, SIN or MTX *P* > 0.05). Noticeably, using the same statistical analysis, ΔDAS28 correlation with delta IL-12/P40 (Figure 8C, SIN *P* = 0.216, MTX *P* = 0.0013) or Eotaxin-2 (Figure 8H, SIN *P* = 0.0254, MTX *P* = 0.059) differed between SIN and MTX. However, a separate ΔDAS28 correlation analysis of the changes in the levels of other 10 cytokines under SIN or MTX treatments showed almost no difference between the two therapeutic strategies.

### SIN reduced the percentage of CD14^+^CD16^+^ monocytes in RA patient PBMCs

**Figure 9:**
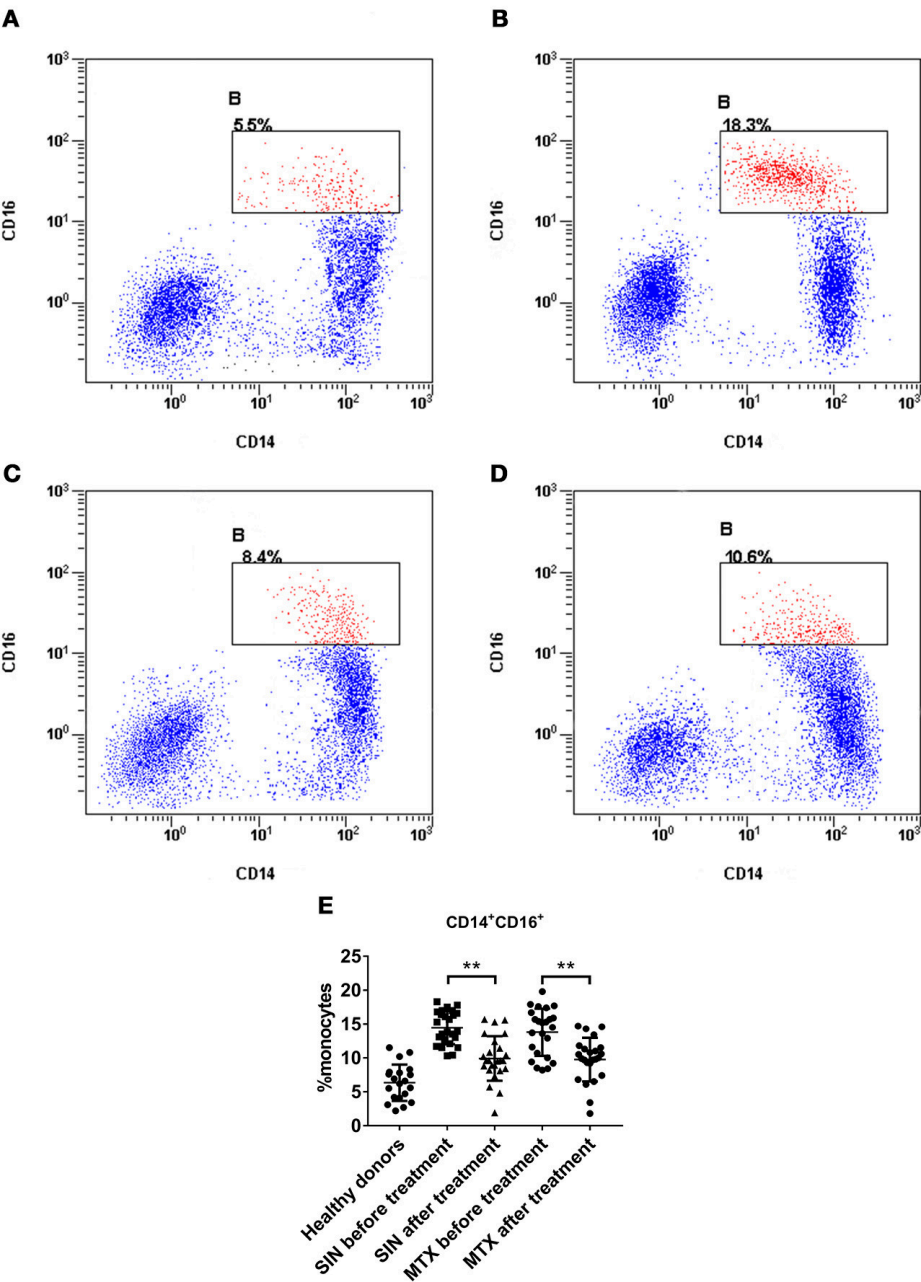
SIN reduced the percentage of CD14^+^CD16^+^ monocytes in RA patient PBMCs. A representative flow cytometry analysis of CD14^+^CD16^+^ percentage in PBMCs of **(A)** heathy donors **(B)** patient with active RA **(C)** after SIN treatment RA patient **(D)** after MTX treatment RA patient. **(E)** Analysis of the percentage of CD14^+^CD16^+^ monocytes in PBMCs of each group. Heathy donors, *n* = 20, SIN before treatment, *n* = 25, SIN after treatment, *n* = 25, MTX before treatment, *n* = 24, MTX after treatment, *n* = 24. Each dot represents one sample. Significance was analyzed by unpaired two-sided *t*-test. **P < 0.01.

CD14^+^CD16^+^ monocytes in PBMCs have been identified as a minor population of monocytes in human peripheral blood (PB). In addition, they have been implicated in several inflammatory diseases including RA (37, 41). They can produce pro-inflammatory cytokines except IL-10, which is secreted by CD16^−^ monocytes. However, we did not find a significant difference in CD16^−^ monocytes among these groups (data was not shown). This population characteristic coincided with the levels of cytokines in RA patients. The percentage of CD14^+^CD16^+^ monocytes in healthy donors was lower than 10% (Figure 9A). The percentage of CD14^+^CD16^+^ in RA patients before therapy was higher than 10% (Figure 9B), but it could be suppressed by SIN (Figure 9C) or MTX (Figure 9D). The percentage of CD14^+^CD16^+^ monocytes in PBMC of all samples was shown in Figure 9E. The baseline mean ± SD frequency of CD14^+^CD16^+^ monocytes was 6.34 ± 2.678% in healthy donors group(*n* = 20). In RA patients before treatment, it was elevated to 14.14 ± 2.999% (*n* = 49). After 3 months treatment, The mean ± SD frequency of CD14^+^CD16^+^ monocytes was suppressed to 9.916 ± 3.284% from 14.47 ± 2.452% under SIN treatment (*n* = 25, ^**^P < 0.05). It was also decreased to 9.754 ± 3.216% from 13.79 ± 3.499% under MTX treatment.(*n* = 24, ^**^P < 0.05).

## Discussion

Rheumatoid arthritis (RA) is a multifactorial, polygenic disease, which can be considered a realistic challenge to the scientific community (42). The various pro-inflammatory cytokines (e.g., IL-1, IL-6, IL-8, TNF-α, and PGE2) and the variety of matrix metalloproteinases secreted by infiltrating macrophages in synovial fluids, which are involved in T cell activation and proliferation and mediation of cell-cell interaction, result in the release of tissue-damaging enzymes, which eventually lead to inflammation propagation and joint damage in RA (8, 43).

Sinomenine (SIN) was found to have anti-rheumatic, pharmacological function in the 1970s (22). Currently, SIN has been approved for RA treatment by the China Food and Drug Administration (CFDA), although the mechanism of its anti-rheumatic effects has not been fully elucidated. Previous studies have reported that SIN has an anti-inflammatory function in adjuvant arthritis rats (26, 28) and that it regulates T cells and Th17 cells in gut-associated lymphoid tissues (44). Moreover, it has been reported to regulate Th1 and Th2 immune responses in mice subcutaneously immunized with ovalbumin (OVA) emulsified with complete Freund's adjuvant (25). Furthermore, SIN could regulate inflammatory cytokines in other inflammatory disease models (45, 46). However, to our knowledge, our study is the first to screen and validate the anti-inflammatory effect of SIN on cytokine secretion *in vivo* and *in vitro*. From *in vitro* studies, we demonstrated that SIN protected RAW264.7 from LPS-induced cell damage and screened 40 cytokines by inflammatory cytokine array and qRT-PCR. Twelve cytokines (IL-6, GM-CSF, IL-12 p40, IL-1α, IL-1β, TNF-α, KC, Eotaxin-2, IL-10, M-CSF, RANTES, and MCP-1) dramatically affected by SIN were selected and authenticated by ELISA. However, together with these 12 cytokines, only nine cytokines were significantly regulated by SIN as assessed by qRT-PCR. These data indicated that SIN did not affect M-CSF, RANTES, and MCP-1 mRNA transcription, but could affect their secretion, possibly through post-transcriptional regulations, such as protein synthesis or extracellular transportation. Interestingly, we found that M-CSF and MCP-1 secretion was slightly decreased by SIN, but no significant change was observed *in vitro*. However, the effects of SIN on their *in vivo* secretion were confirmed. We speculated that this due to the fact that the LPS-induced cell model did not represent inflammation progression completely. After all, multiple cell types and metabolic pathways are involved in inflammation and immune regulation *in vivo*.

Many linkage studies in humans and in animal models of RA, in particular, murine collagen induced arthritis (CIA), have consistently shown that there are many quantitative trait loci (QTL) contributing to disease susceptibility (47). The data from the CIA mouse model demonstrated that 100 mg/kg/day SIN administration could achieve good remission in CIA mice, which was consistent with other previous researches (44). More importantly, the secretion of 11 cytokines was suppressed, whereas IL-10 secretion was induced by SIN in CIA mice; this was a strong evidence proving that SIN has an anti-inflammatory function, which was consistent with the *in vitro* data.

SIN also possesses favorable curative effects in terms of clinical applications. Meta-analysis to compare the efficacy and safety of SIN preparations with those of NSAIDs concluded that using SIN for the clinical treatment of RA might be more favorable than NSAIDs (48). Interestingly, these findings are consistent with our previous systematic review about safety and efficacy of SIN compared to MTX in RA treatment (24). Our present study focused on cytokine changes before and after SIN or MTX treatment and the correlation between their changes and clinical remission score, DAS28. The cytokine secretion data collected from RA patients demonstrated that SIN could regulate the secretion of 12 cytokines validated in RAW264.7 and mouse model. IL-6, GM-CSF, IL-12 p40, IL-1α, IL-1β, TNF-α, GROα, Eotaxin-2, and IL-10 D-value before and after treatment correlated with delta DAS28. However, there were three cytokines—M-CSF, RANTES, MCP-1—whose D-values had no obvious correlation with delta DAS28. These data were consistent with a previous study (49). We speculated that these three cytokines were not the determinants of altered DAS28. Of course, it might be because our sample size was so limited that it could not sufficiently reflect the correlation between them. Nevertheless, it was encouraging that, overall, 12 validated cytokines responded to SIN treatment in varying degrees.

The pathogenesis of RA does not reflect the action of any single cell lineage, but rather the complex interactions between all cell populations in the RA synovium, mediated by both direct cell-cell contact and by molecules that are secreted or shed by the various types of synovial cells (14). It has been reported that within the synovial environment in RA, the proportion of multiple cell populations that produce various cytokines are very high (50). Monocyte/macrophage subsets are particularly important in RA progression besides cytokines because monocyte activation or macrophage recruitment is critical step in RA. Monocytes/macrophages are a potent source of pro-inflammatory cytokines and these cells can also produce a wide range of chemokines, which help recruit additional leukocytes to the inflamed joint (51). As such, monocytes and macrophages are viewed as relevant therapeutic targets in RA (52, 53). Although the heterogeneous populations of macrophages in RA have not been fully characterized, preliminary results in arthritis mouse models have identified the phenotype and ontogeny of synovial macrophages and deciphered the properties of monocyte-derived infiltrating and tissue-resident macrophages (54).

In the present study, we detected two kinds of monocytes/macrophages in the CIA mouse model and CD14^+^CD16^+^ monocytes in RA patients before and after SIN or MTX treatment. The results from a number of studies have directly or indirectly proved that Ly6C^+^ monocytes cause autoimmune arthritis in the CIA model, while the exact contribution of Ly6C^−^ monocytes remains unclear (17, 54). CD43 was a novel redefined marker of inflammatory monocytes in murine spleen (36). The F4/80 molecule was established as a unique marker of murine macrophages when a monoclonal antibody was found to recognize an antigen exclusively expressed by these cells. However, besides F4/80, additional molecules must also be examined to distinguish these cells from other immune cells according to a recent study (55). Therefore, CD64 was suggested as a marker of M1-type dysregulated macrophages (56). CD14^+^CD16^+^ was identified as an increased subset of PBMCs in RA or inflammation disorders (37, 41). As shown in the results, SIN exerted a strong mediating ability to regulate monocyte/macrophage populations, which may possibly explain why it could affect the secretion of multiple cytokines. Of course, cytokine regulation and monocyte/macrophage population regulation could happen simultaneously. Cytokines and monocytes/macrophages could stimulate each other and form feedback loop. Apparently, SIN effectively repressed this loop in RA progression.

Indeed, a plethora of conventional synthetic disease-modifying anti-rheumatic drugs (csDMARDs), biological DMARDs (bDMARDs), and targeted synthetic DMARDs (tsDMARDs) can be used in different sequences and/or combinations to treat RA patients (57). However, combination therapy with csDMARDs was not superior to MTX monotherapy, and monotherapy was better tolerated (58). Moreover, MTX therapy for patients with RA is accompanied by a variety of changes in serum cytokine expression, which in turn correlates strongly with clinical disease activity (59). Thus, in the present study, to reduce the confounding factor to the uttermost during the evaluation of SIN therapeutic effects on early diagnosed RA patients, MTX monotherapy was taken as a positive remission drug for comparison.

Inflammatory cytokines are the center of the complex inflammatory networks that propagate and perpetuate RA (60). Cytokine inhibitors, also known as biologicals, have been definitively proved to play a critical role in TNF-α and IL-6 in disease pathogenesis and possibly also for GM-CSF (61). With new biologicals in the pipeline and different formulations of established compounds, treatment options for RA will become even more versatile and sophisticated (62). In particular, drugs such as Ustekinumab, an anti-IL-12/23 p40 mAb, and Guselkumab (CNTO 1959), a compound targeting IL-23 p19 (63) may be used for RA treatment. Small molecule inhibitors targeting the JAK pathway are a promising new treatment strategy for RA. Tofacitinib (JAK1/3 inhibitor) and Baricitinib (JAK1/2 inhibitor) are being developed to further interrogate the potential of JAK family proteins as effective targets (64). However, there are still some barriers or risks limiting the application of these biologicals. Undeniably, standard-dose and high-dose biologicals (with/without traditional DMARDs) are associated with an increase in serious infections compared to those with/without traditional DMARDs in RA, while low-dose biologics are not implicated in causing infections (57, 65). Especially, cost-effectiveness was also an issue of concern regarding bDMARDs for both rheumatologists and patients. Rheumatologists are willing to trade among treatment efficacy, patient treatment preferences, and economic considerations (66). Actually, SIN is a cost-effective therapeutic strategy for RA treatment. Over the years, most of the conventional drugs that have been used for RA treatment have severe adverse reactions and are quite expensive. Natural plant products comprise one of the most popular complementary and alternative medicines for inflammatory and immune disorders (67). Based on the present study, we believe that SIN can serve as a multiple cytokine inhibitor similar to the single targeted biologicals mentioned above. Furthermore, it could be used to treat other inflammatory diseases in addition to RA.

There were some limitations to this study. First, the mechanism of SIN anti-rheumatic effect was not fully elucidated. We could not determine the entire target that could be affected by SIN. The regulatory network of SIN is yet to be explored. In addition, considering the ethical requirements, we collected limited clinical samples, and these patients were early diagnosed RA patients. RA is a chronic disease that can persist for decades. Therefore, the effectiveness and safety of SIN need to be evaluated for longer periods. Moreover, to verify the current evidence from this study, further trials with a high-quality study design should be conducted and more long-term study data from clinical trials will need to be systematically assessed in the future.

## Ethics statement

This study was carried out in accordance with the recommendations of EULAR recommendations for the management of rheumatoid arthritis with synthetic and biological disease-modifying antirheumatic drugs: 2016 update, European League Against Rheumatism (EULAR). The protocol was approved by the Medical Ethics Committee of Nanjing Hospital of Chinese Medicine. All subjects gave written informed consent in accordance with the Declaration of Helsinki.

## Author contributions

WL and YZ did the cell experiments, cytokines screening, ELISA, clinical data collecting, wrote the manuscript. WZ did the flow cytometry analysis. CM did the animal experiment. JR did the qRT-PCR and statistical analysis. HL and YW designed the study and provided funding support.

### Conflict of interest statement

The authors declare that the research was conducted in the absence of any commercial or financial relationships that could be construed as a potential conflict of interest.

## Supplementary material

Figure S1 Experimental design workflow. Step 1, Secreted cytokines screening in four groups: untreated control, LPS (1 μg/mL), SIN (10 μg/mL) + LPS (1 μg/mL), SIN (50 μg/mL) + LPS (1 μg/mL). Step 2, mRNA screening in the four groups above. Step 3, different expressed cytokines were validated by ELISA *in vitro* and *in vivo*. Step 4, different expressed cytokines were validated in human serum samples by ELISA. Step 5, Statistical analysis of the correlation between the different expression levels of cytokines and clinical indexes. χ^2^-test evaluated the therapeutic effects of MTX and SIN on RA.

Figure S2 Screening data of cytokine induced by LPS or its co-incubation with SIN in RAW264.7. RAW 264.7 cells (1.0 × 10^7^ cells/100 mm dish) were pre-treated with different concentrations (0, 10, 50 μg/mL) for 2 h, and co-stimulated with 1 μg/mL of LPS for another 24 h. At the end of the incubation period, RAW 264.7 serum-free supernatants were collected and assayed for cytokine production using the Mouse Inflammation Antibody Array C1. **(A)** Each cytokine is represented by duplicate spots in the location shown. **(B)** Scanning cytokine spots corresponding to chart above. **(C,D)** The relative multiple cytokine level of each cytokine spot on the chip. Values are presented as mean ± S.D. *n* = 4, ^*^*P* < 0.05 vs. untreated control; ^#^*P* < 0.05 vs. LPS treated RAW264.7. The detailed significant difference is shown in Table S1.

Figure S3 Relative mRNA levels of the 40 cytokines in the arrays were determined using RT-PCR. The results of RT-PCR were normalized to β-actin and expressed as fold change to untreated control. The values represent the means ± SD of triplicate experiments. ^*^*P* < 0.05 vs. LPS treated RAW264.7.

Figure S4 Typical FACS analysis of **(A)** synovial, **(B)** spleen and **(C)** ankle macrophage subpopulations in 100 mg/kg/day SIN group.

Figure S5 Paired comparisons of DAS28 in RA patients before or after treatment with SIN or MTX. **(A)** SIN treatment **(B)** MTX treatment. The Wilcoxon paired signed rank test was used to compare clinical score. SIN, *n* = 25; MTX, *n* = 24; ^**^*P* < 0.01, ^***^*P* < 0.001.

Table S1 Primers used in RT-PCR.

Table S2 ELISA Kit used for validation of cell culture supernatants and human serum samples.

Table S3 Antibodies used in the immune cell subset detection by flow cytometry.

Table S4 Detailed screening data of cytokine array.

## Abbreviations

RA: rheumatoid arthritis
SIN: sinomenine
CIA: collagen-induced arthritis
qRT-PCR: quantitative real-time polymerase chain reaction
DAS28: disease activity score of 28 joints
TNF-α: tumor necrosis factor alpha
LPS: lipopolysaccharide
MTX: methotrexate
NSAIDs: non-steroidal anti-inflammatory drugs
DMARD: disease modifying anti-rheumatic drug
csDMARDs: conventional synthetic disease-modifying anti-rheumatic drugs
bDMARDs: biological DMARDs
tsDMARDs: targeted synthetic DMARDs
ADEs: adverse events
FTN: Zhengqing Fengtongning
PBMCs: peripheral blood mononuclear cells
PB: peripheral blood
HPLC: high-performance liquid chromatography
FBS: fetal bovine serum
PBS: phosphate buffer saline
DMEM: Dulbecco's modified eagle's medium
CCK-8: cell counting kit-8
ELISA: enzyme-linked immunosorbent assay
ATCC: American type culture collection
IFA: incomplete Freund's adjuvant
TJC: tender joint count
SJC: swollen joint count
CCP: cyclic citrullinated peptide antibody
ESR: erythrocyte sedimentation rate
CRP: C-reactive protein
IFX: infliximab
ADA: adalimumab
ETN: etanercept
CZP: certolizumab
GLM: golimumab
TNFi: tumor necrosis factor inhibitors
GM-CSF: granulocyte–macrophage colony-stimulating factor.
