# Supplementary figures and images for: Sinomenine Inhibits the Progression of Rheumatoid Arthritis by Regulating the Secretion of Inflammatory Cytokines and Monocyte/Macrophage Subsets
Source: Front Immunol. 2018 Sep 26;9:2228. doi: 10.3389/fimmu.2018.02228 (PMC6168735; doi:10.3389/fimmu.2018.02228)

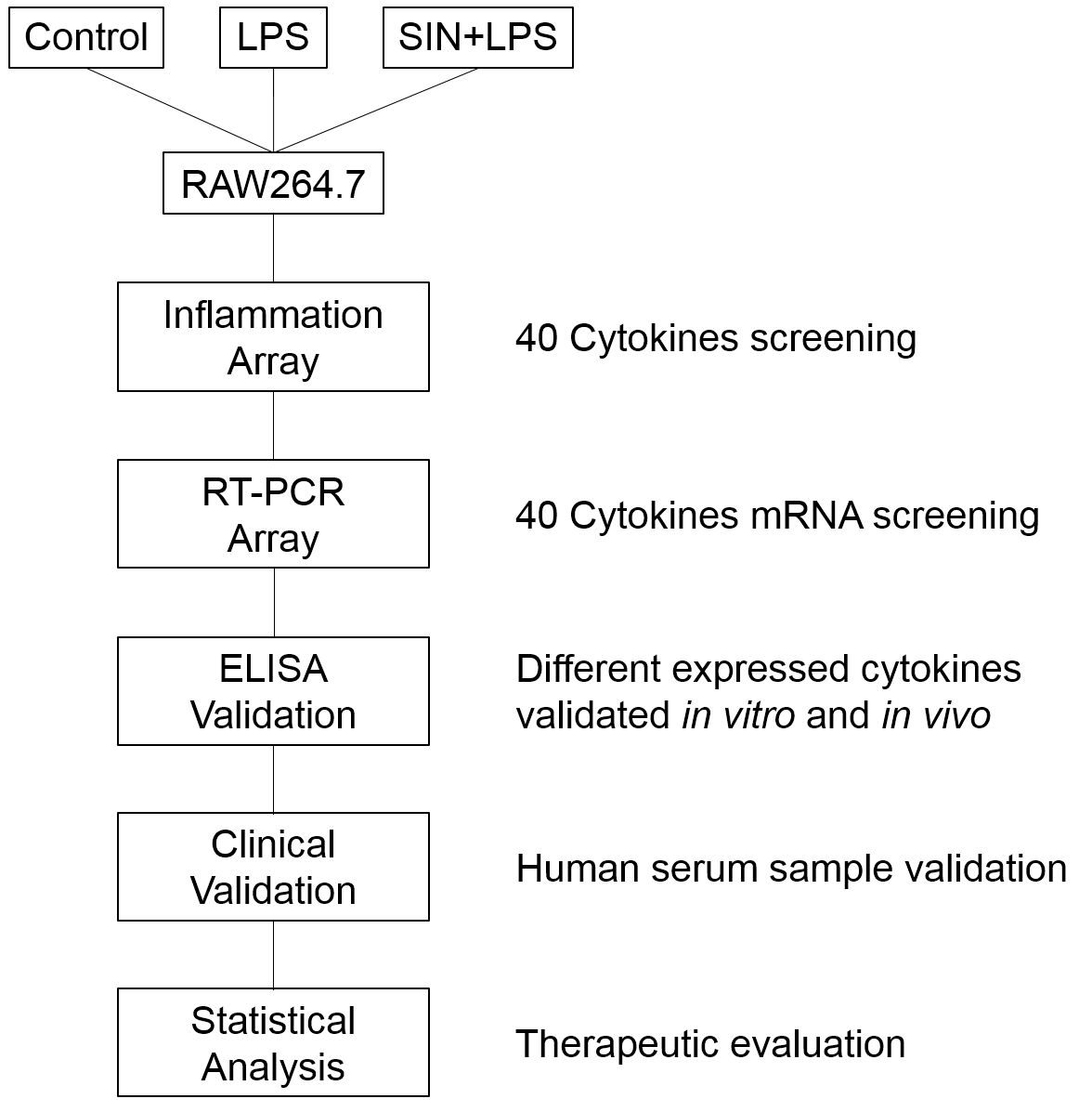

Supplement: Figure S1 — Experimental design workflow. Step 1, Secreted cytokines screening in four groups: untreated control, LPS (1 μg/mL), SIN (10 μg/mL) + LPS (1 μg/mL), SIN (50 μg/mL) + LPS (1 μg/mL). Step 2, mRNA screening in the four groups above. Step 3, different expressed cytokines were validated by ELISA in vitro and in vivo. Step 4, different expressed cytokines were validated in human serum samples by ELISA. Step 5, Statistical analysis of the correlation between the different expression levels of cytokines and clinical indexes. χ2-test evaluated the therapeutic effects of MTX and SIN on RA. [file Image_1.TIF]

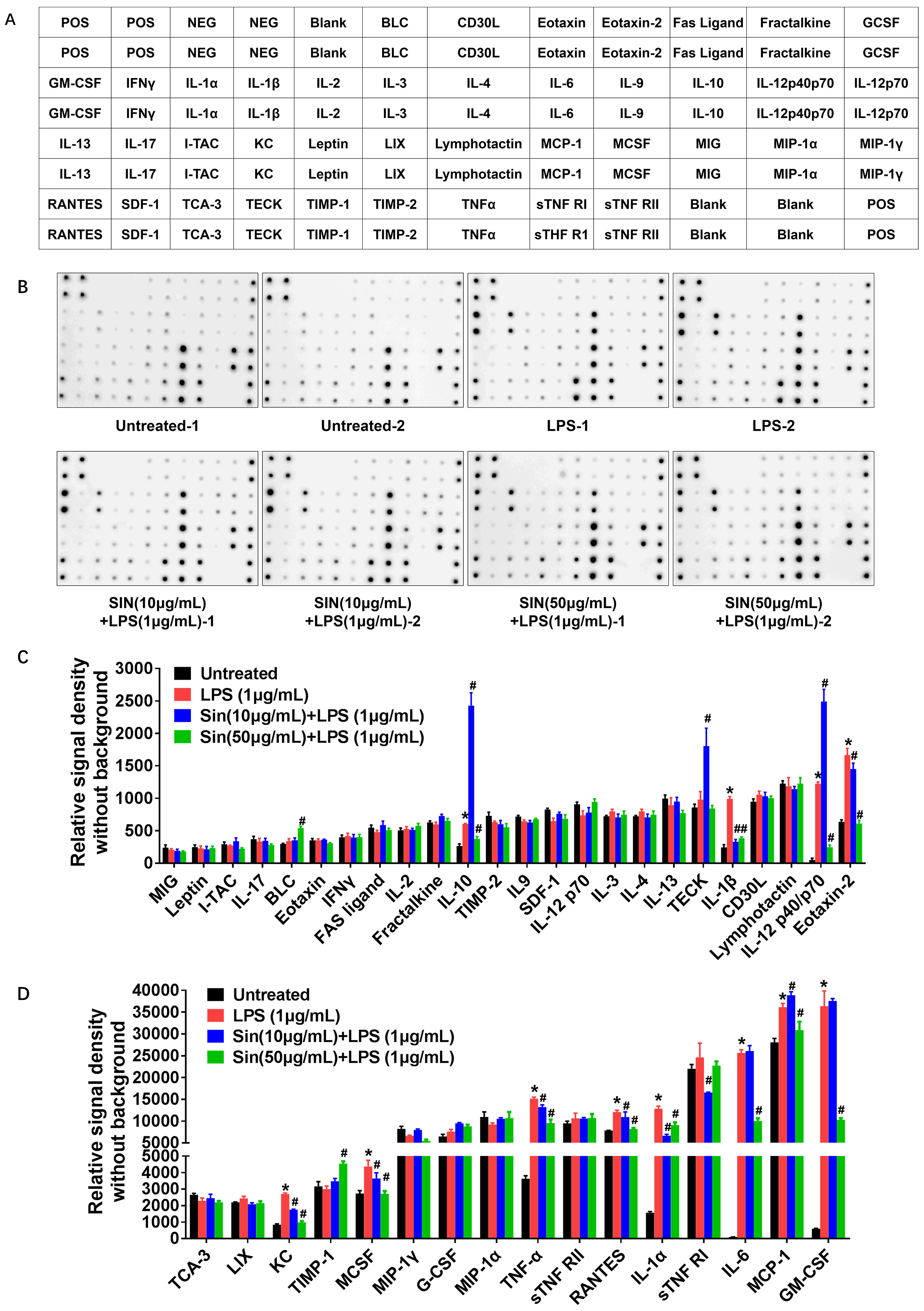

Supplement: Figure S2 — Screening data of cytokine induced by LPS or its co-incubation with SIN in RAW264.7. RAW 264.7 cells (1.0 × 10^7^ cells/100 mm dish) were pre-treated with different concentrations (0, 10, 50 μg/mL) for 2 h, and co-stimulated with 1 μg/mL of LPS for another 24 h. At the end of the incubation period, RAW 264.7 serum-free supernatants were collected and assayed for cytokine production using the Mouse Inflammation Antibody Array C1. (A) Each cytokine is represented by duplicate spots in the location shown. (B) Scanning cytokine spots corresponding to chart above. (C,D) The relative multiple cytokine level of each cytokine spot on the chip. Values are presented as mean ± S.D. n = 4, *P < 0.05 vs. untreated control; #P < 0.05 vs. LPS treated RAW264.7. The detailed significant difference is shown in Table S1. [file Image_2.TIF]

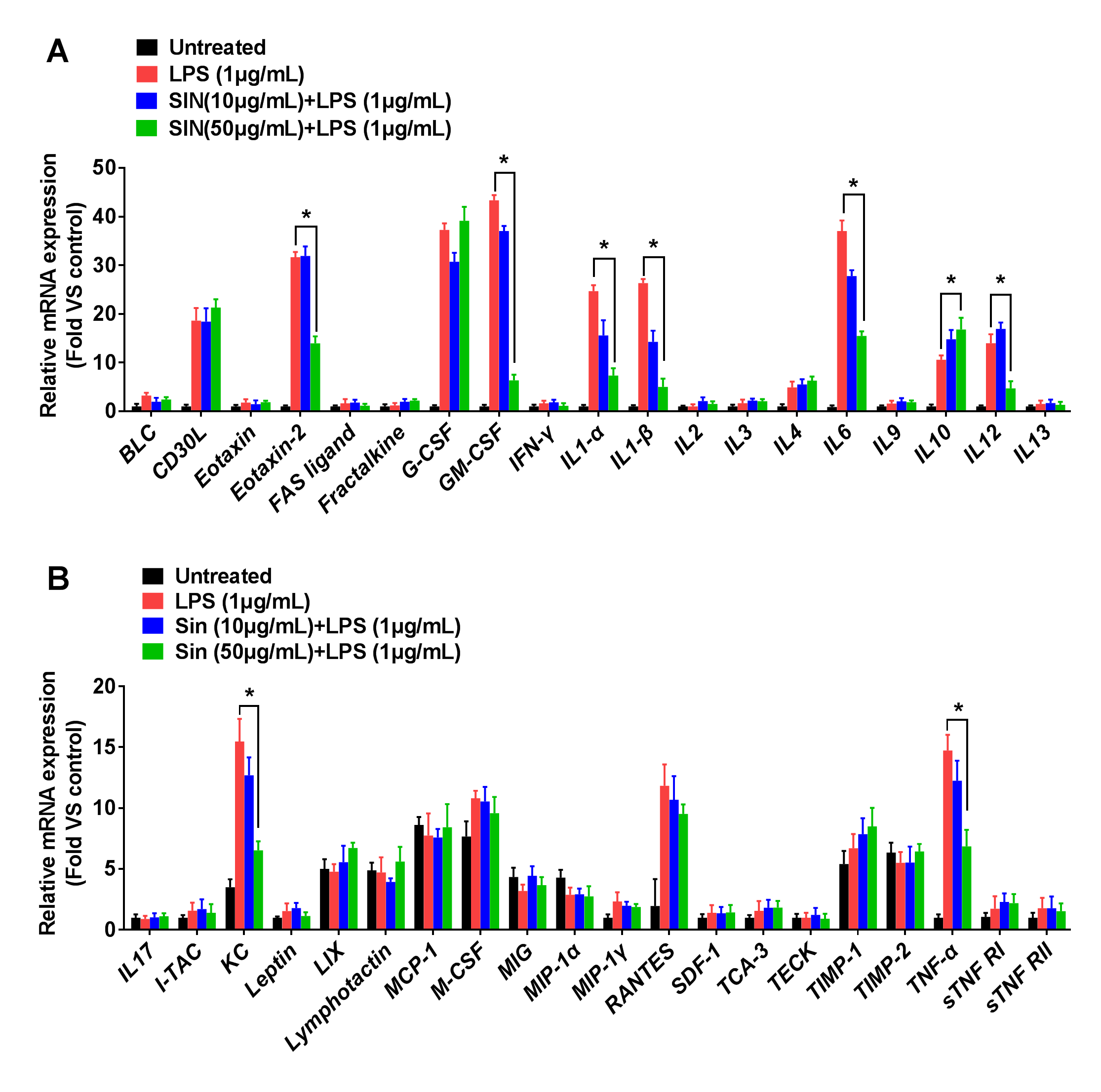

Supplement: Figure S3 — Relative mRNA levels of the 40 cytokines in the arrays were determined using RT-PCR. The results of RT-PCR were normalized to β-actin and expressed as fold change to untreated control. The values represent the means ± SD of triplicate experiments. *P < 0.05 vs. LPS treated RAW264.7. [file Image_3.TIF]

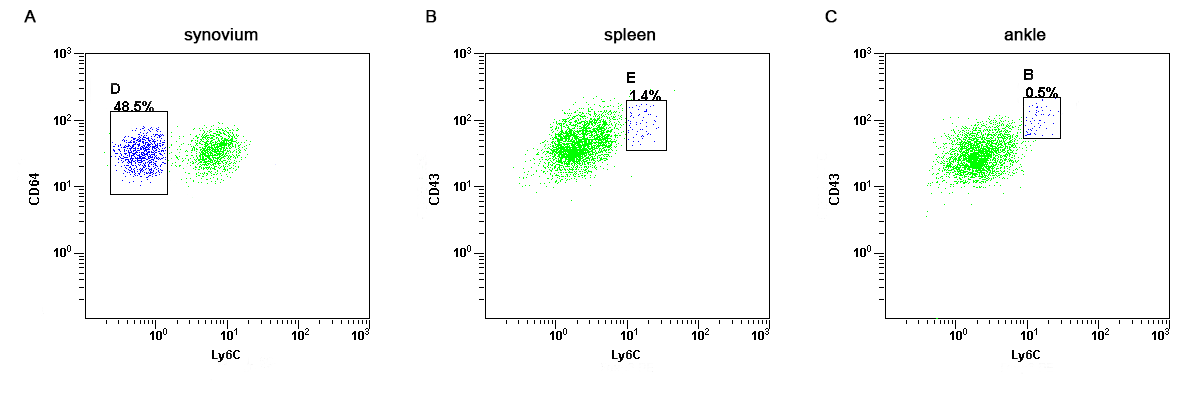

Supplement: Figure S4 — Typical FACS analysis of (A) synovial, (B) spleen and (C) ankle macrophage subpopulations in 100 mg/kg/day SIN group. [file Image_4.TIF]
