# Supplementary material for: Sinomenine Inhibits the Progression of Rheumatoid Arthritis by Regulating the Secretion of Inflammatory Cytokines and Monocyte/Macrophage Subsets
Source: Front Immunol. 2018 Sep 26;9:2228. doi: 10.3389/fimmu.2018.02228 (PMC6168735; doi:10.3389/fimmu.2018.02228)
Supplement: Table S1 — Primers used in RT-PCR. [file Table_1.DOCX]

**Supplementary materials and methods**

Table S1

Primers used in RT-PCR

| **Gene ID** | **PrimerName** | **Sequence** |
| --- | --- | --- |
| 55985 | BLC(CXCL13)-F | GGCCACGGTATTCTGGAAGC |
|  | BLC(CXCL13)-R | ACCGACAACAGTTGAAATCACTC |
| 21949 | CD30 Ligand(TNFSF8)-F | GCAGCTACTTCTACCTCAGCA |
|  | CD30 Ligand(TNFSF8)-R | GCCATCTTCGTTCCATGACAGT |
| 20292 | Eotaxin-1(CCL11)-F | GAATCACCAACAACAGATGCAC |
|  | Eotaxin-1(CCL11)-R | ATCCTGGACCCACTTCTTCTT |
| 56221 | Eotaxin-2(MPIF-2/CCL24)-F | TCTTGCTGCACGTCCTTTATT |
|  | Eotaxin-2(MPIF-2/CCL24)-R | GCATCCAGTTTTTGTATGTGCC |
| 14102 | Fas Ligand(TNFSF6)-F | TATCAAGGAGGCCCATTTTGC |
|  | Fas Ligand(TNFSF6)-R | TGTTTCCACTTCTAAACCATGCT |
| 20312 | Fractalkine(CX3CL1)-F | ACGAAATGCGAAATCATGTGC |
|  | Fractalkine(CX3CL1)-R | CTGTGTCGTCTCCAGGACAA |
| 12985 | GCSF-F | ATGGCTCAACTTTCTGCCCAG |
|  | GCSF-R | CTGACAGTGACCAGGGGAAC |
| 12981 | GM-CSF-F | GGCCTTGGAAGCATGTAGAGG |
|  | GM-CSF-R | GGAGAACTCGTTAGAGACGACTT |
| 15978 | IFN-gamma-F | ATGAACGCTACACACTGCATC |
|  | IFN-gamma-R | CCATCCTTTTGCCAGTTCCTC |
| 16175 | IL-1 alpha(IL-1 F1)-F | CGAAGACTACAGTTCTGCCATT |
|  | IL-1 alpha(IL-1 F1)-R | GACGTTTCAGAGGTTCTCAGAG |
| 16176 | IL-1 beta(IL-1 F2)-F | GCAACTGTTCCTGAACTCAACT |
|  | IL-1 beta(IL-1 F2)-R | ATCTTTTGGGGTCCGTCAACT |
| 16183 | IL-2-F | TGAGCAGGATGGAGAATTACAGG |
|  | IL-2-R | GTCCAAGTTCATCTTCTAGGCAC |
| 16187 | IL-3-F | GGGATACCCACCGTTTAACCA |
|  | IL-3-R | AGGTTTACTCTCCGAAAGCTCTT |
| 16189 | IL-4-F | GGTCTCAACCCCCAGCTAGT |
|  | IL-4-R | GCCGATGATCTCTCTCAAGTGAT |
| 16193 | IL-6-F | TAGTCCTTCCTACCCCAATTTCC |
|  | IL-6-R | TTGGTCCTTAGCCACTCCTTC |
| 16198 | IL-9-F | ATGTTGGTGACATACATCCTTGC |
|  | IL-9-R | TGACGGTGGATCATCCTTCAG |
| 16153 | IL-10-F | GCTCTTACTGACTGGCATGAG |
|  | IL-10-R | CGCAGCTCTAGGAGCATGTG |
| 16159 | IL-12 p40(12B)/p70(12A)-F | CTGTGCCTTGGTAGCATCTATG |
|  | IL-12 p40(12B)/p70(12A)-R | GCAGAGTCTCGCCATTATGATTC |
| 16163 | IL-13-F | CCTGGCTCTTGCTTGCCTT |
|  | IL-13-R | GGTCTTGTGTGATGTTGCTCA |
| 16171 | IL-17A-F | TTTAACTCCCTTGGCGCAAAA |
|  | IL-17A-R | CTTTCCCTCCGCATTGACAC |
| 56066 | I-TAC(CXCL11)-F | GGCTTCCTTATGTTCAAACAGGG |
|  | I-TAC(CXCL11)-R | GCCGTTACTCGGGTAAATTACA |
| 14825 | KC(CXCL1)-F | CTGGGATTCACCTCAAGAACATC |
|  | KC(CXCL1)-R | CAGGGTCAAGGCAAGCCTC |
| 16846 | Leptin-F | GAGACCCCTGTGTCGGTTC |
|  | Leptin-R | CTGCGTGTGTGAAATGTCATTG |
| 20311 | LIX-F | TCCAGCTCGCCATTCATGC |
|  | LIX-R | TTGCGGCTATGACTGAGGAAG |
| 16963 | Lymphotactin(XCL1)-F | TTTGTCACCAAACGAGGACTAAA |
|  | Lymphotactin(XCL1)-R | CCAGTCAGGGTTATCGCTGTG |
| 20296 | MCP-1(CCL2)-F | TTAAAAACCTGGATCGGAACCAA |
|  | MCP-1(CCL2)-R | GCATTAGCTTCAGATTTACGGGT |
| 12977 | M-CSF-F | ATGAGCAGGAGTATTGCCAAGG |
|  | M-CSF-R | TCCATTCCCAATCATGTGGCTA |
| 17329 | MIG(CXCL9)-F | TCCTTTTGGGCATCATCTTCC |
|  | MIG(CXCL9)-R | TTTGTAGTGGATCGTGCCTCG |
| 20302 | MIP-1 alpha(CCL3)-F | TTCTCTGTACCATGACACTCTGC |
|  | MIP-1 alpha(CCL3) | CGTGGAATCTTCCGGCTGTAG |
| 20308 | MIP-1 gamma-F | CCCTCTCCTTCCTCATTCTTACA |
|  | MIP-1 gamma-R | AGTCTTGAAAGCCCATGTGAAA |
| 20304 | RANTES(CCL5)-F | GCTGCTTTGCCTACCTCTCC |
|  | RANTES(CCL5)-R | TCGAGTGACAAACACGACTGC |
| 20315 | SDF-1 alpha(CXCL12 alpha)-F | TGCATCAGTGACGGTAAACCA |
|  | SDF-1 alpha(CXCL12 alpha)-R | TTCTTCAGCCGTGCAACAATC |
| 20315 | I-309(TCA-3/CCL1)-F | GGCTGCCGTGTGGATACAG |
|  | I-309(TCA-3/CCL1)-R | AGGTGATTTTGAACCCACGTTT |
| 20300 | TECK(CCL25)-F | TTACCAGCACAGGATCAAATGG |
|  | TECK(CCL25)-R | CGGAAGTAGAATCTCACAGCAC |
| 21857 | TIMP-1-F | GCAACTCGGACCTGGTCATAA |
|  | TIMP-1-R | CGGCCCGTGATGAGAAACT |
| 21858 | TIMP-2-F | TCAGAGCCAAAGCAGTGAGC |
|  | TIMP-2-R | GCCGTGTAGATAAACTCGATGTC |
| 21926 | TNF alpha-F | CCCTCACACTCAGATCATCTTCT |
|  | TNF alpha-R | GCTACGACGTGGGCTACAG |
| 21937 | TNF RI(TNFRSF1A)-F | CCGGGAGAAGAGGGATAGCTT |
|  | TNF RI(TNFRSF1A)-R | TCGGACAGTCACTCACCAAGT |
| 21938 | TNF RII(TNFRSF1B)-F | ACACCCTACAAACCGGAACC |
|  | TNF RII(TNFRSF1B)-R | AGCCTTCCTGTCATAGTATTCCT |
