# Supplementary material for: Sinomenine Inhibits the Progression of Rheumatoid Arthritis by Regulating the Secretion of Inflammatory Cytokines and Monocyte/Macrophage Subsets
Source: Front Immunol. 2018 Sep 26;9:2228. doi: 10.3389/fimmu.2018.02228 (PMC6168735; doi:10.3389/fimmu.2018.02228)
Supplement: Table S2 — ELISA Kit used for validation of cell culture supernatants and human serum samples. [file Table_2.DOCX]

**Supplementary materials and methods**

Table S2

ELISA Kit used in validation of cell culture supernatants and human serum samples

| ELISA Kit | Company | Address | CODE | Catalog# |
| --- | --- | --- | --- | --- |
| Mouse TNF-alpha | Raybiotech | Norcross, GA, USA | ELM-TNFa-1 | ELM-TNFa |
| Mouse IL-6 | Raybiotech | Norcross, GA, USA | ELM-IL6-1 | ELM-IL6 |
| Mouse GM-CSF | Raybiotech | Norcross, GA, USA | ELM-GMCSF-1 | ELM-GMCSF |
| Mouse M-CSF | Raybiotech | Norcross, GA, USA | ELM-MCSF | ELM-MCSF |
| Mouse IL-12 p40/p70 | Raybiotech | Norcross, GA, USA | ELM-IL12P40P70-1 | ELM-IL12P40P70 |
| Mouse Eotaxin-2 | Raybiotech | Norcross, GA, USA | ELM-Eotaxin2-1 | ELM-Eotaxin2 |
| Mouse IL1-alpha | Raybiotech | Norcross, GA, USA | ELM-IL1a-1 | ELM-IL1a |
| Mouse IL1-beta | Raybiotech | Norcross, GA, USA | ELM-IL1b-1 | ELM-IL1b |
| Mouse MCP-1 | Raybiotech | Norcross, GA, USA | ELM-MCP1-1 | Mouse MCP1 |
| Mouse KC(CXCL1) | Raybiotech | Norcross, GA, USA | ELM-KC-1 | ELM-KC |
| Mouse RANTES | Raybiotech | Norcross, GA, USA | ELM-RANTES-1 | ELM-RANTES |
| Mouse MCP-1 | Raybiotech | Norcross, GA, USA | ELM-MCP1-1 | ELM-MCP1 |
| Human TNF-alpha | Raybiotech | Norcross, GA, USA | ELH-TNFa-1 | ELH-TNFa |
| Human IL-6 | Raybiotech | Norcross, GA, USA | ELH-IL6-1 | ELH-IL6 |
| Human GM-CSF | Raybiotech | Norcross, GA, USA | ELH-GMCSF-1 | ELH-GMCSF |
| Human M-CSF | Raybiotech | Norcross, GA, USA | ELH-MCSF-1 | ELH-MCSF |
| Human Eotaxin-2 | Raybiotech | Norcross, GA, USA | ELH-Eotaxin2-1 | ELM-Eotaxin2-1 |
| Human IL1-alpha | Raybiotech | Norcross, GA, USA | ELH-IL1a-1 | ELH-IL1a |
| Human IL1-beta | Raybiotech | Norcross, GA, USA | ELH-IL1b-1 | ELH-IL1b |
| Human IL-10 | Raybiotech | Norcross, GA, USA | ELH-IL10-1 | ELH-IL10 |
| Human IL-12 p40 | Raybiotech | Norcross, GA, USA | ELH-IL12p40-1 | ELH-IL12p40 |
| Human RANTES | Raybiotech | Norcross, GA, USA | ELH-RANTES-1 | ELH-RANTES |
| Human MCP-1 | Raybiotech | Norcross, GA, USA | ELH-MCP1-1 | ELH-MCP1 |
| Human GRO-alpha (CXCL1) | Raybiotech | Norcross, GA, USA | ELH-GROa-1 | ELH-GROa |
