# Supplementary material for: Sinomenine Inhibits the Progression of Rheumatoid Arthritis by Regulating the Secretion of Inflammatory Cytokines and Monocyte/Macrophage Subsets
Source: Front Immunol. 2018 Sep 26;9:2228. doi: 10.3389/fimmu.2018.02228 (PMC6168735; doi:10.3389/fimmu.2018.02228)
Supplement: Table S3 — Antibodies used in the immune cell subset detection by flow cytometry. [file Table_3.DOCX]

**Supplementary materials and methods**

Table S3

Antibodies used in the immune cell subsets detection by flow cytometry

| Antibody | Label | Clone | Isotype Control | Company |
| --- | --- | --- | --- | --- |
| CD11b | FITC | M1/70 | Rat IgG2b,κ | eBioscience |
| F4/80 | APC | BM8 | Rat IgG2a, κ | Biolegend |
| CD64 | APC/Cy7 | X54-5/7.1 | Mouse IgG1, κ | Biolegend |
| Ly6C | APC | HK1.4 | Rat IgG2c, κ | Biolegend |
| CD43 | APC/Cy7 | 1B11 | Rat IgG2a, κ | Biolegend |
| CD14 | FITC | 63D3 | Mouse IgG1, κ | Biolegend |
| CD16 | PE | 3G8 | Mouse IgG1, κ | Biolegend |
